# Morphological profiling by high-throughput single-cell biophysical fractometry

**DOI:** 10.1038/s42003-023-04839-6

**Published:** 2023-04-24

**Authors:** Ziqi Zhang, Kelvin C. M. Lee, Dickson M. D. Siu, Michelle C. K. Lo, Queenie T. K. Lai, Edmund Y. Lam, Kevin K. Tsia

**Affiliations:** 1 Department of Electrical and Electronic Engineering, The University of Hong Kong, Pokfulam, Hong Kong; 2 Advanced Biomedical Instrumentation Centre, Hong Kong Science Park, Shatin, New Territories Hong Kong

**Keywords:** High-throughput screening, Optical imaging

## Abstract

Complex and irregular cell architecture is known to statistically exhibit fractal geometry, i.e., a pattern resembles a smaller part of itself. Although fractal variations in cells are proven to be closely associated with the disease-related phenotypes that are otherwise obscured in the standard cell-based assays, fractal analysis with single-cell precision remains largely unexplored. To close this gap, here we develop an image-based approach that quantifies a multitude of single-cell biophysical fractal-related properties at subcellular resolution. Taking together with its high-throughput single-cell imaging performance (~10,000 cells/sec), this technique, termed single-cell biophysical fractometry, offers sufficient statistical power for delineating the cellular heterogeneity, in the context of lung-cancer cell subtype classification, drug response assays and cell-cycle progression tracking. Further correlative fractal analysis shows that single-cell biophysical fractometry can enrich the standard morphological profiling depth and spearhead systematic fractal analysis of how cell morphology encodes cellular health and pathological conditions.

## Abstract (web summary)

A high-throughput image-based approach quantifies single-cell biophysical fractal-related properties at subcellular resolution with statistical power for cell classification, drug response assays, and cell-cycle progression tracking.

## Introduction

Cell morphology is constituted by the complex biomolecular machinery at the genomic, transcriptomics, and proteomic levels. Hence, it is a valuable readout, which can be captured by microscopy, for assaying the functional state of individual cells. Dramatic advancements in high-throughput imaging and computer vision in the past decade have sparked the major drive in the use of microscopy to extract quantifiable information from cell morphology (i.e., morphological profiling)^1,2^. Creating the catalogs of the cell morphological features, this cell profiling strategy enables mining the underlying feature signatures or patterns that can infer cell age^3^, metastatic potential^4^, screening chemical^5^, and genetic perturbation^6^.

In morphological profiling, a wealth of quantitative metrics (features) can be extracted from the individual cell images, including cell size, shape, texture etc., representing a fingerprint of each cell. Downstream analysis is then applied to investigate the similarities or correlations between profiles in order to identify the phenotypes specific to the cell types and states. Traditionally, the morphological features are defined based on Euclidean geometry, which can be easily coupled with general variations in geometry (e.g., size and shape), however, irregular spatial information hidden in the complex cellular structure (e.g., statistical properties of shape and texture) could often be missed. This is particularly relevant to the cellular malignancy, in which the intracellular mass growth shows a significant degree of randomness and disorder^7^. Specifically, conventional Euclidean geometry fails to holistically quantify the textural or shape irregularity at different length scales in simple words, due to the metrical variation along with the change of measurement resolution. This explains the need for an extended set of local and global features to separately examine the heterogeneity at different spatial scales^8^. Yet, they do not capture an important property shared in a wide variety of biological cells, i.e., “fractality”. It refers to the fact that the texture pattern/shape of an object does not significantly differ from the same property measured on a larger scale. To this end, fractal dimension (FD) has been adopted as an effective metric that quantifies and classifies the irregular biological structures and the self-similarity characteristics that are not well represented by the Euclidean geometry^9^. FD is typically a non-integer value, in contrast to the dimensions defined in Euclidean geometry, i.e., 1 for a line (1D), 2 for a plane (2D), and 3 for a cube (3D).

Indeed, fractal analysis has been demonstrated as an effective tool in clinical diagnosis, such as the examination of aberrant histopathological features in tissues^10^, and assessment of abnormal organ morphology (e.g. tumor vasculatures) in radiology^11^. Further down to the cellular or even subcellular level, fractal behavior can be observed in the chromatin topology in the nucleus^12–14^, cell membrane contour and adhesion topology^15^, mitochondrial^16^, and cytoskeleton^17^ morphology. For instance, the mitochondria organization undergoing fission and fusion regulated by cellular metabolism follows the statistics of self-similarity^18^; the protein interaction and structural interminglement of chromatin are both highly consistent with a fractal framework^12^; the architecture of cytoskeleton and plasma membrane also shows a self-similar topology where molecules are confined hierarchically over time and length scales^19^. Hence, the knowledge of fractal characteristics at the single-cell level could offer new physical insights into cell types and states different from the tissue’s fractal properties. Specifically, changes in cellular and subcellular FDs are now known to be closely related to the epigenetic states^20^, the gene expression levels^16^. Hence, they can be indicative of the functional states of cells (e.g., metabolic states^21^, cell differentiation states^22^, and cell malignancy^23^).

However, these promises, together with the cellular fractal characteristics that can readily be analyzed by standard microscopy, have not yet made fractal analysis widely applicable in cytometry and morphological profiling of cells. The key challenge stems from the fact that the cellular/subcellular morphology exhibits fractal properties in a statistical sense, instead of the archetypal geometrical sense. Yet, current imaging techniques lack the scale and throughput to guarantee that fractal analysis could show the sufficient statistical power for delineating the cellular heterogeneity and complexity on the single-cell level (e.g., limited to ~10’s −100’s single cells^15,23,24^).

To address this challenge, here we employ an ultrahigh-throughput quantitative phase imaging (QPI) flow cytometer called multiplexed asymmetric-detection time-stretch optical microscopy (multi-ATOM)^25,26^ to analyze single-cell biophysical fractal characteristics (termed *single-cell biophysical fractometry*) at the breadth and depth not achievable by the existing methods. This strategy of single-cell fractometry is achieved by two key attributes: (1) Establishing label-free morphological profiling that includes not only the common shape and texture features based on Euclidean geometry, but also a collection of biophysical fractal parameters (not only FD) of each cell. This is enabled by the core strength of QPI inherited by multi-ATOM, in which the *complex-field* image information of individual cells (i.e., both amplitude and quantitative phase images) can be obtained at subcellular resolution. Such complex-field information can then be harnessed to compute the corresponding far-field light-scattering pattern (by means of Fourier Transform light-scattering (FTLS)^27^), which provides a catalog of single-cell fractal and the associated ALS (angular light-scattering) features. Defining these fractal features as an intrinsic morphological profile aligns precisely with the growing interest in new strategies for in-depth biophysical phenotyping of cells, which has already generated new mechanistic knowledge of cell heterogeneity and showed initial promises in identifying cost-effective biomarkers of disease, thanks to its label-free nature^28^. (2) Enabling large-scale single-cell fractometry by the ultrafast QPI operation in multi-ATOM, at the speed at least 100 times faster than the existing QPI modalities that rely on camera technology for image recording^29^. We note that another form of QPI, digital holographic imaging, has also recently been employed to perform fractal analysis of non-biological microparticles and microalgae at a moderate throughput^30,31^. Combined with the high-throughput microfluidics platform^8,25,26,32^, this approach enables single-cell fractometry at a throughput of at least 10,000 cells/sec without sacrificing the subcellular imaging resolution. This attribute critically provides in-depth statistical fractal analysis, which has largely been underexploited in the previous work on fractal analysis, especially at the single-cell screening resolution. Indeed, comprehensive morphological profiling often relies on analyzing the deeper statistics of cell phenotypes in order to detect cellular heterogeneity and subpopulation with an improved sensitivity and robustness^33^. More importantly, in order gain a better interpretation of the significance of the extracted fractal-related features, we further investigate the underlying correlation, if any, between Euclidean-defined morphological features and the fractal features.

In this work, we show that high-throughput single-cell biophysical fractometry allows us to distinguish the histological subtypes of lung cancer cell lines by fractal-related features. We also demonstrate that these fractal-related features can be used for assessing drug treatment responses. These features also play an important role in identifying different stages of cell cycle progression (G1, S, and G2).

## Results

### Key workflow and basic performance of single-cell biophysical fractometry

**Fig. 1:**
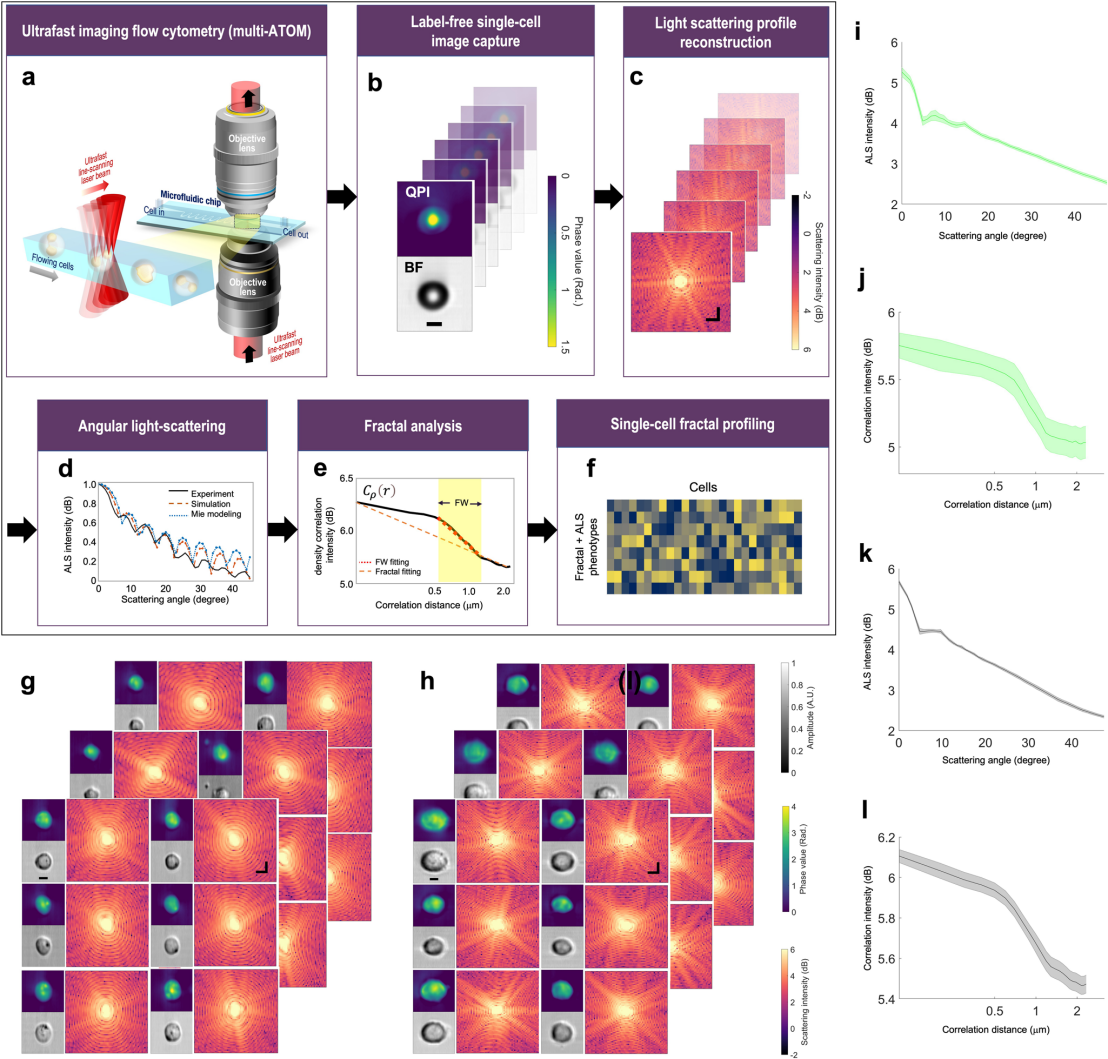
General workflow and performance of high-throughput single-cell fractal profiling. **a** Ultrafast imaging flow cytometry by multi-ATOM. **b** Label-free single-cell image capture (Top: QPI; bottom: Bright-field image (BF)) (Example: polymer microspheres). Scale bar = 5 μm. **c** Complex-field light scattering profile reconstruction via FTLS (Example: polymer microspheres). Scale bar = 2 rad/μm. **d** Retrieval of the ALS profile. (Example: Experimental microsphere data acquired by multi-ATOM (red) and theoretical simulation result (blue) and the analytical Mie light scattering theory of ideal sphere with the same size as the spheres (black). **e** Schematics of fractal fitting of ${C}_{\rho }\left(r\right)$. FD is determined from the fractal fitting of the overall curve, whereas the fitting within FW gives FD with FW. **f** ALS and fractal profiles extracted from (**d**, **e**). **g** Label-free single-cell image capture of leukemia cell line (ACC220). Spatial scale bar = 5 μm. Frequency scale bar = 2 rad/μm. **h** Label-free single-cell image capture exampled by leukemia cell line (THP-1). Spatial scale bar = 5 μm. Frequency scale bar = 2 rad**/**μm. **i** ALS plots summarizing ALS profiles of 2500 ACC220 cells. Shaded area indicates the statistical variance. **j** Plot summarizing ${C}_{\rho }\left(r\right)$ of 2,500 ACC220 cells. Shaded area indicates the statistical variance. **k** ALS plot summarizing ALS profiles of 2500 THP-1 cells. Shaded area indicates the statistical variance. **l** Plot summarizing ${C}_{\rho }\left(r\right)$ of 2500 THP-1 cells. Shaded area indicates the statistical variance.

We applied multi-ATOM, an ultrafast QPI modality, to perform high-throughput single-cell imaging in microfluidic flow (See Methods) (Fig. 1a). Detailed working principle and experimental configuration were reported previously^8,25,26^. The general principle is to first record the complex optical field at the image plane of the flowing cell by multi-ATOM, i.e., $E\left(x,y\right)=A\left(x,y\right){{{{{{\rm{e}}}}}}}^{j\phi \left(x,y\right)}$, where $A\left(x,y\right)$ is amplitude (i.e. bright-field) image and $\phi \left(x,y\right)$ is the quantitative phase image (Fig. 1b). Subsequently, the complex field at the image plane is then numerically propagated to the far field using the Fourier transform operation—yielding the (far-field) scattered light-field pattern $S({k}_{x},{k}_{y})=\,{{{{{\mathcal{F}}}}}}[E(x,y)]$^27^, from which the fractal properties of the cell can be measured (Fig. 1c). It is due to the fact that the cellular and subcellular fractal structures give rise to the heterogeneity of the refractive index within the cells, and thus directly impact the scattered light pattern^34^. Here, we further convert the scattered light pattern into an angular light scattering (ALS) profile $S\left(q\right)$ in which scattered light intensity is averaged over rings of constant wave vector $q=4{{{{{\rm{\pi }}}}}}/\lambda {{\sin }}(\theta /2)$, where $\theta$ is the *polar* scattering angle^27^ (Fig. 1d). This approach has been adopted in characterizing different metabolic states of red blood cells (RBCs)^35^, assessing different intracellular organelles^36^, and classification of bacterial species^37^, and analyzing the fractal characterization of the fibrin network^38^, all in a label-free manner.

Based on the light scattering theory^39^, we can further relate the ALS with the dry mass density variation $\rho (r)$ (quantified through spatial correlation of the density fluctuation ${C}_{\rho }(r)$)(See Methods)^40^ in such a way that the Fourier transform of an ALS intensity profile will obey an inverse power law relationship, i.e., ${{{{{\mathcal{F}}}}}}\left[{\left|S\left(\theta \right)\right|}^{2}\right]\propto {C}_{\rho }\left(r\right)\propto {r}^{-\alpha }$, where $\alpha$ is the exponent, and ${C}_{\rho }\left(r\right)$ is the spatial correlation of $\rho (r)$. In practice, by fitting the slope $\alpha$ of the log-scaled plot of ${{{{{\mathcal{F}}}}}}\left[{\left|S\left(\theta \right)\right|}^{2}\right]$, we could calculate the FD = $3-\alpha$ (See detailed derivation in Methods) (Fig. 1e). As cells exhibit fractal properties (e.g., self-similarity) only within a limited range of length scales, referred to as the fractal window (FW), ALS offers a powerful tool to identify the FW where the inverse power law behavior is present^21,41^. In addition, as mentioned earlier, the fractal behaviors of cells are manifested in a quantitative statistical sense. We exploited a catalog of parameters that quantify the statistics of the ALS profiles, and the statistics related to FW fitting, e.g., the mean square error (MSE), the FW width, and the estimated FD (See the complete list of parameters in Supplementary Table S1) (Fig. 1f). Here FW is defined as the interval of correlation distance in ${C}_{\rho }\left(r\right)$ with the most prominent downwards slope (See Supplementary Table S1). We note that these FD-related parameters can reflect how well the fractality is preserved in different scales and allow us to quantify the degree of self-similarity. The basic performance of imaging and the ALS analysis were tested with microbeads (Fig. 1b–d), and two different types of leukemic cells, ACC220 and THP-1 (Fig. 1g–l).

### Single-cell fractal profiles show differences among lung cancer cell subtypes

Cell morphology assessment is commonly practiced in cancer diagnosis and classification. Taking lung cancer, the leading cause of cancer-related mortality worldwide^42^, as an example, histological characterizations of small biopsies or cytology specimens play an integral role in the pipeline for classifying different lung cancer types, according to the criteria long-established by World Health Organization (WHO)^43,44^. However, these assessments are often confounded by subjective and biased visual inspection and are mostly limited to obvious morphological abnormalities across the histochemically stained tissues, e.g., cell shapes and intercellular textural complexity. As abnormal subcellular morphology (e.g. nucleus/nucleoli and cytoplasm) is also found to be indicative of malignancy and different cancer subtypes^45,46^, we sought to investigate if the single-cell fractal properties of different lung cancer cell subtypes extracted from the label-free ALS profiles can provide unbiased classification of the three key different lung cancer subtypes which are commonly categorized through histopathological examination: small cell lung carcinoma (SCLC) and two subtypes of non–small cell lung carcinoma (NSCLC), which are squamous cell carcinoma (SCC) and adenocarcinoma (ADC)^43^.

**Fig. 2:**
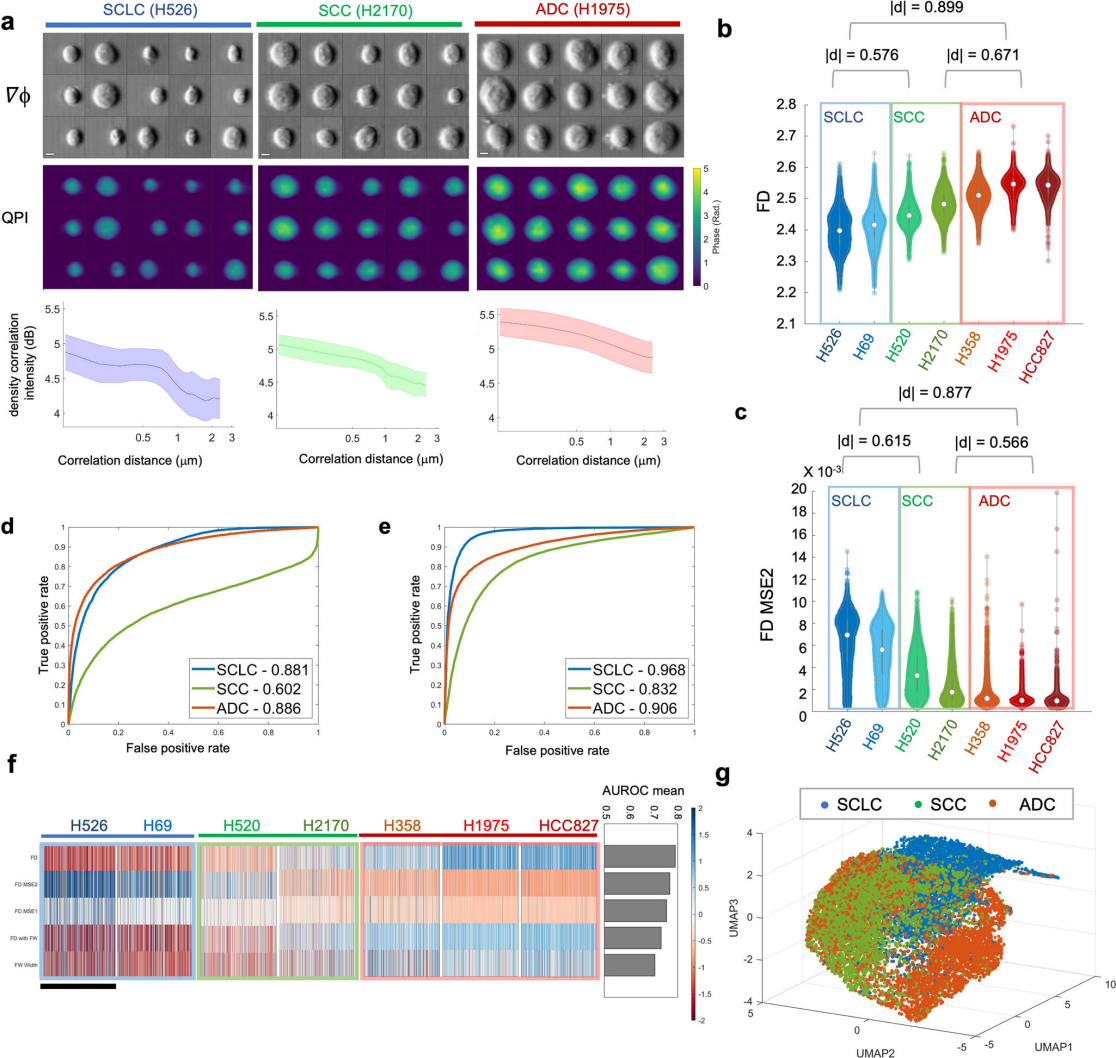
Fractometry of multiple lung cancer cell subtypes. **a** Randomly selected phase gradient images (∇ϕ_x_, top), QPI (middle) and the correlation function plots (bottom) of three lung cancer cell lines, respectively. Scalar bar is 5 μm. Shaded area indicates the statistical variance. **b** Statistical distributions of FD. **c** Statistical distributions of FD MSE2. **d**, **e** ROC curves for distinguishing the lung cancer subtypes in one-versus-all mode with **d** FD only and **e** the linear regression classifiers constructed from all the 17-dimension fractal and ALS features. AUROC values are labeled in the legend. **f** The fractal phenotypic profile of 7 lung cancer cell lines (feature values are normalized based on the *z* score). Each row represents a fractal feature, and each column represents a single cell. The scale bar stands for 1000 cells, which were randomly subsampled from each cell line. The AUROC ranking results are shown in the right panel. **g** 3D UMAP visualization of the 17 fractal and ALS-related phenotypes extracted from single-cell images of the lung cancer cell lines. Clusters are colored according to the three lung cancer subtypes.

Based on the visual examination of the randomly selected reconstructed single-cell phase gradient and quantitative phase images captured by multi-ATOM (Fig. 2a), it is generally impractical to distinguish the 3 subtypes by standard bulk features such as cell size and optical density (or opacity). Because of the heterogeneity among individual cells, the morphological difference across subtypes is challenging to define using only one or a few phenotypes. For instance, although the general size distribution of the population differs between SCLC and NSCLC, there is a significant size variation on the single-cell level even within the same subtype. Therefore, characterization that depends solely on these bulk features alone, including cell size, is not an effective way for label-free single-cell identification between the heterogenous populations of SCLC and NSCLC. To augment the dimensions of our morphological profiling, we parameterized the cell morphology (recorded by multi-ATOM) through high-order statistical analysis, which reveal global and local textural features of the cells^8^. Going beyond, we evaluated the ${C}_{\rho }\left(r\right)$ from the ALS profile ($S\left(\theta \right)$) of individual cells (Fig. 2a). We observed that ${C}_{\rho }\left(r\right)$ appears to be statistically different among 3 subtypes (including a total of 7 different lung cancer cell lines (Supplementary Fig. S1)).

Henceforth, we calculated the FD of individual cells through inverse power law fitting and observed that the FD distributions can relatively be categorized into the low/middle/high level for SCLC, SCC, and ADC, respectively (Fig. 2b), with a significant effect size ($\left|d\right|$ ~0.57–0.89 between subtypes using Cliff’s delta statistics). The statistics of the overall fitting error (FD MSE2) also shows significant difference among the subtypes ($\left|d\right|$~ 0.57–0.88). More importantly, FD MSE2 indicates that there is a larger variance (or dispersion) of ${C}_{\rho }\left(r\right)$ for SCLC, while the cells of SCC and ADC both tend to keep a better linearity, implying the self-similarity of cellular structure is more consistently preserved in a wider length scale (Fig. 2c). We stress that effect size, which is independent of sample size, is adopted here to evaluate the significance of sample difference. This is due to the fact that the common *p*-value will give misleading high statistical significance when using a large sample size^47^, which is challenging to achieve in other fractal cellular measurements but realized by our multi-ATOM system (>10,000 cells). Besides effective size analysis, we also computed the Spearman correlation coefficient of fractal features and lung cancer subtype (1 for SCLC, 2 for SCC, and 3 for ADC) to prove their close bonding (FD: 0.7174; FD MSE2: −0.6791).

We also studied the feasibility of using fractal features to distinguish the three lung cancer subtypes. Based on one-versus-all classification, we computed their area-under-curve of the receiver operating characteristics (AUROC). We observed that using FD alone can already allow us to distinguish the main groups of NSCLC from SCLC, with the reasonably high accuracy (AUROC score of 0.881). FD-only classification of the two subtypes of NSCLC (i.e. ADC and SCC) is found to be more challenging (AUROC = 0.602). (Fig. 2d). To test further, we extracted the statistics of other fractal- and ALS-related features from ${C}_{\rho }\left(r\right)$ (17 dimensions in total, see Supplementary Table S1) to construct the linear regression classifiers. We observed that the classification accuracy was enhanced noticeably for all three subtypes (particularly for SCC) according to the ROC analysis (AUROC ~0.832—0.968 (Fig. 2e)). These results illustrate that fractal features have adequate effectiveness in the categorization of lung cancer subtypes, in addition to providing meaningful physical interpretation regarding cell complexity.

Leveraging the statistical power offered by multi-ATOM, we further generated a 2D heatmap to display the single-cell fractal profile (Fig. 2f), where each column represents the normalized profile of an individual cell and each row represents a subsampling set of the referring feature (1000 cells were randomly picked for each cell line). Based on the heatmap of the five most significant fractal features (ranked by the mean AUROC of three one-versus-all subtype classification) (right panel of Fig. 2f), we observe that each of the three lung cancer subtypes displays its distinct characteristic pattern in the fractal profile. For instance, the three ADC cell lines (H358, H1975, HCC827) share a similar profile that shows high FD, low FD MSE2, and high FD width. When we further included other ALS features for higher-dimensional analysis, visualized by the uniform manifold approximation and projection (UMAP) algorithm^48^ (Fig. 2g), we observed the three main clusters corresponding to the key lung cancer subtypes. Although overlapping is observable to some degree in the clusters of ADC and SCC, most cells of SCLC are highly dispersed from NSCLC. Hence, this study suggests that morphological profiling based on these label-free single-cell fractal features, which are closely linked to the intracellular mass density distribution characteristics, could provide the discriminative power to distinguish the key histologically different lung cancer subtypes.

### Single-cell fractal profiles encodes cellular responses to drug treatments

**Fig. 3:**
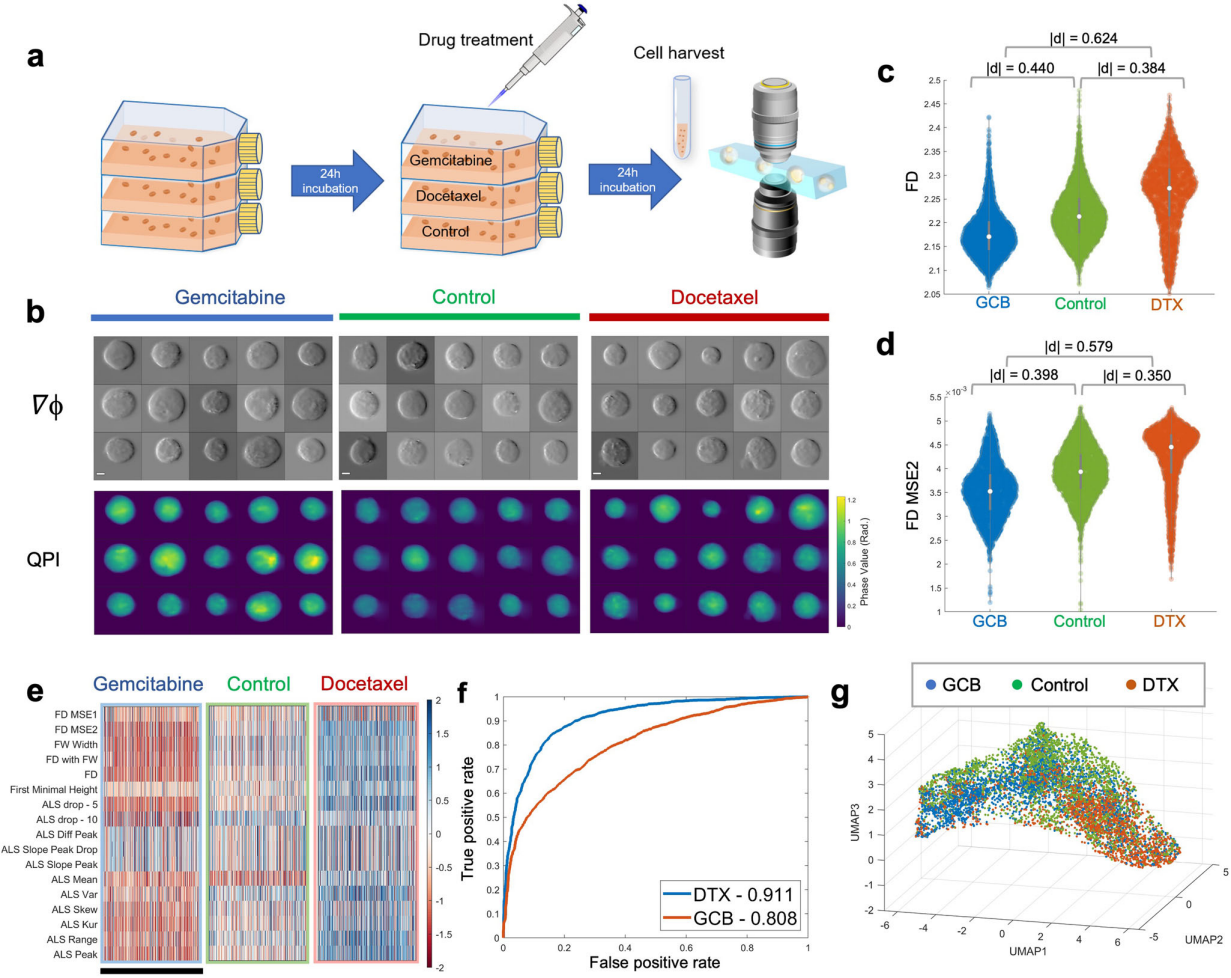
Single-cell biophysical fractometry for drug response analysis of H2170 cells. **a** Experimental workflow. **b** Randomly selected phase gradient images (∇ϕ_x_, top), QPI (bottom) of H2170 cells treated with DTX, control and GCB, respectively. Scalar bar is 5 μm. **c** Statistical distributions of FD. **d** Statistical distributions of FD MSE2. **e** A fractal phenotypic profile of three different drug treatment (feature values are normalized based on the z-score). Each row represents a fractal feature, and each column represents a single cell. The scale bar stands for 1000 cells, which were randomly subsampled from each group. **f** ROC curves of the linear regression classifiers differing DTX/GCB-treated cells from control group, respectively, which were constructed from 17-dimension Fourier features. AUROC values are labeled in the legend. **g** 3D UMAP visualization of the ALS-related phenotypes extracted from single-cell images of drug-treated H2170 cells. Dispersion among different drug-treated clusters is observable.

Going beyond cell-type classification, we next validated the sensitivity of this refractometry profiling in response to the drug treatment of with different mechanisms of action, in order to demonstrate its potential applications in label-free drug screening. We acquired massive single-cell images of squamous cell carcinoma (H2170) cells by multi-ATOM imaging flow cytometry. The cells were treated with two different drugs at their respective IC_50_ (half maximal inhibitory concentration, see Methods for details) for 24 h (Fig. 3a). The two drugs were Docetaxel (DTX), which is a microtubule stabilizer inducing cell-cycle arrest and death^49^, and Gemcitabine(GCB), which is an analog of deoxycytidine inhibiting DNA synthesis and elongation and thus causing DNA fragmentation and apoptotic cell death of malignant cells^50^. Over 2000 images of each set were collected for feature extraction, from which we observed the changes in cell morphologies in response to the drug treatments (Fig. 3b).

We subsequently evaluated the statistical distributions of the primary fractal features (Fig. 3c, d), and found that both FD and FD MSE2 exhibit a significant decrease ($\left|d\right|$ = 0.440 and 0.398, respectively) when GCB was applied, whereas present a sizable increase ($\left|d\right|$ = 0.384 and 0.350) under the addition of DTX. The distinguishable response of the three sets indicates that cells treated with GCB are in better conformity with fractal pattern, but possess less complex subcellular structures. This can be attributable to the inhibited DNA replication by GCB. GCB mainly interferes the cell growth by triggering chain termination after blending into the elongating DNA strands. This mechanism results in a reduced complexity of individual cells (smaller FD) and a less local morphological/mass variation without active DNA synthesis (lower MSE). On the other hand, DTX promotes the assembly of microtubules and prevents their dynamic polymerization/depolymerization^51^, which is required for the mitosis-related functions (e.g., formation of spindle, binding to mitochondria and chromosomes). Therefore, DTX blocks cell division after chromosomes are duplicated, leading to a higher cell morphological complexity (higher FD). A larger MSE also suggests that the fractal pattern of the DTX-treated cells is less well-fit than that of the control group, indicating a more prominent difference across multiple fractal architectures of intracellular structures.

Moreover, using similar approach utilized in the previous section of lung cancer analysis, a heatmap combining all 17-dimension fractal and ALS features can be generated to portray the fractal-related profile of cells in response to the drug treatment (Fig. 3e). In general, we note that most of the characteristics of GCB-treated cells have a lower value, but show an increase in the DTX group, yet both of them differ considerably from the control group in the presented pattern. To further quantify the significance of this difference, we again constructed two linear regression classifiers to distinguish the drug-treated groups and control group with the 17-dimension Fourier features, respectively. The corresponding ROC test results in an AUROC value of over 0.80 to both drugs. Notably, the discrimination power to GCB treatment reaches 0.911 (Fig. 3f). Meanwhile, high-dimensional visualization of ALS features via UMAP also showed that the ALS profiling of cells treated with different drugs had observable dispersed distribution and formed disparate clusters (Fig. 3g). All of the findings above demonstrated that not only can our fractal profiling strategy accurately identify the changes of cell morphology under drug treatment, but also provide more granular statistics to investigate the mechanisms of action of various drugs. Hence, single-cell biophysical fractometry could be an effective approach for label-free drug response analysis and screening.

### Single-cell fractal profiles recapitulate cell-cycle progression

**Fig. 4:**
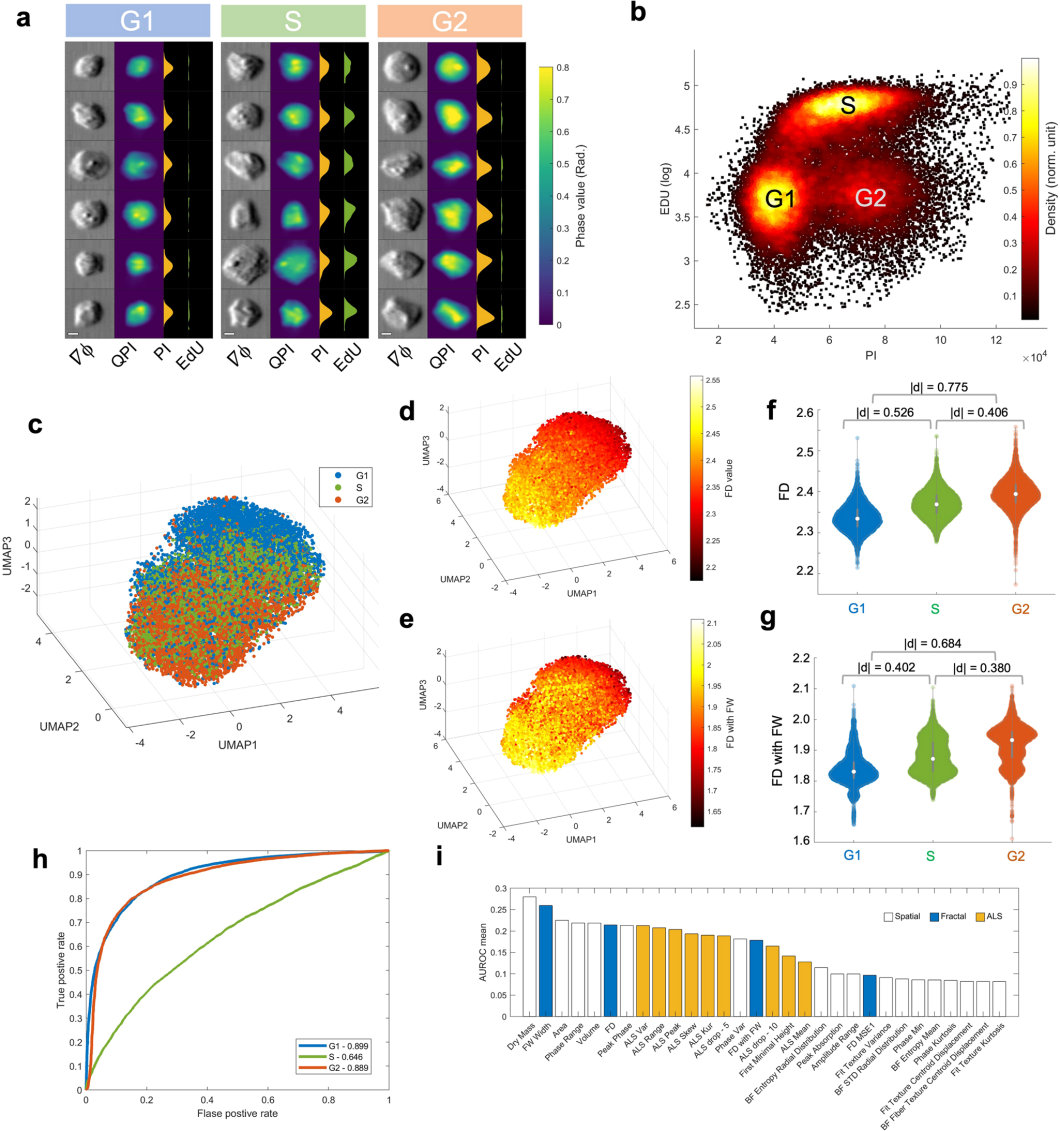
Fractal analysis of cell cycle progression (MB231 cells). **a** Randomly selected single-cell images (Phase gradient ($\nabla \phi$) and QPI) and the synchronized fluorescence detection of the same cells in different cell cycle phases (G1, S, G2): (From left to right), $\nabla \phi$ images, QPI images, fluorescence profile of PI, and fluorescence profile of EdU. Scale bar = 5 μm. **b** Synchronized 2-color (EdU versus PI) fluorescence detection in multi-ATOM. DNA content is quantified by PI intensity, whereas the S-phase cells are recognized by EdU (in log scale). A standard flow cytometry result for cell cycle determination is shown in Supplementary Fig. S3 for reference. **c** 3D UMAP visualization of the full set of phenotypes (both Fourier and spatial) showing the trend of cell cycle progression. **d**, **e** The same UMAP plot in **c** color-coded with the FD value, the FW value, respectively. **f**, **g** Violin plots of FD, and FD with FW, respectively, across the G1, S, and G2 phase. **h** ROC curves to identify the cell cycle phase in one-versus-all mode with the linear regression classifier constructed from all the Fourier features. AUROC values are labeled in the legend. **i** Feature ranking by AUROC mean (Top 30 features).

We further sought to investigate if and how the label-free single-cell fractal characteristics are impacted by different cell states in the cell-cycle progression. In this study, the single-cell QPI/BF image recording of over 15,000 fixed breast cancer cells (MDA-MB231) is synchronized with 2-color fluorescence detection (Fig. 4a, and see Methods). This multimodality allows us to correlate the label-free fractal properties with the DNA content quantified by the fluorescence labels, at the single-cell precision, as the ground truth of cell-cycle progression from G1, S to G2 phase (by the propidium iodide (PI) label which quantifies DNA content whereas EdU (5-ethynyl-2’-deoxyuridine) indicates the newly synthesized DNA in S-phase cells) (Fig. 4b). We note that the variation in the DNA content, and thus the changes in the biophysical properties (including the fractal characteristics) reflects the continuous progression of cells, instead of discrete states of G1, S, and G2^52^. Hence, the “ground truth” given by the 2-color cell-cycle fluorescence markers/labels should cautiously be treated as the reference, which allows us to interpret the biophysical properties (especially FD) based on the established biochemical signatures (e.g., DNA synthesis and replication) and the related biological events (e.g., cell growth and protein synthesis).

To further harness the strength of information-rich morphological profiling, we defined an extensive set of multi-faceted label-free morphological readouts, encompassing the spatial features (directly computed from QPI and BF following a hierarchical strategy^8^), the light-scattering characteristics (extracted from ALS profile), as well as the fractal profile. We observed that this label-free profile revealed the overall trajectory of the cell-cycle progression from G1, S to G2 phase in the UMAP visualization (Fig. 4c). Importantly, the expression variations of the key fractal features, such as FD and FD with FW, also consistently follow the progression (Fig. 4d, e), and show significant differences across the three phases ($\left|d\right| \, > \, 0.40$ for FD; $\left|d\right| \, > \, 0.38$ for FD with FW) (Fig. 4f, g). FD exhibits a progressive increase along the G1-S-G2 order (Fig. 4f), which is consistent with the trend shown by cell size and cell mass (Supplementary Fig. S2), as the size enlargement and mass accumulation are common biophysical traits during cell cycle progression. This suggests a growing complexity and irregularity of intracellular mass distribution, which could be attributable to DNA replication and the subsequent protein synthesis process (e.g. microtubule production) as the cell evolves from the G1, S to G2 phase.

We further assessed the discrimination power to identify the cell cycle phase of fractal features through ROC analysis in a one-versus-all mode. While using FD alone is found to be not sensitive to detect the S-phase, this single fractal feature is on the other hand effective in identifying the G1 and G2 phases, with the AUROC of 0.822 and 0.790 for G1 and G2 phase, respectively. As S phase is a transit state between G1 and G2, it is acceptable that more features are needed for an accurate identification. Therefore, we performed the same ROC analysis with a linear regression classifier integrating all the features extracted from Fourier domain (17 dimensions), and found an improvement of the AUROC for all three phases (G1:0.899, S:0.646, G2:0.889), especially for S phase (Fig. 4h). By performing the same ROC analysis including all the features extracted from QPI/BF morphology the ALS features and the fractal profile (Supplementary Fig. S4), we also quantified the significance of these features in performing the (one-versus-all) classification of the cell-cycle phases (Fig. 4i). We observed that, apart from the cell size and cell mass, which are known to be tightly linked to cell-cycle progression, multiple fractal and ALS features (e.g., FW width and FD) extracted from the FTLS analysis are among the top 30 features with the highest averaged AUROC, which implicates the informativeness of these Fourier-domain features.

**Fig. 5:**
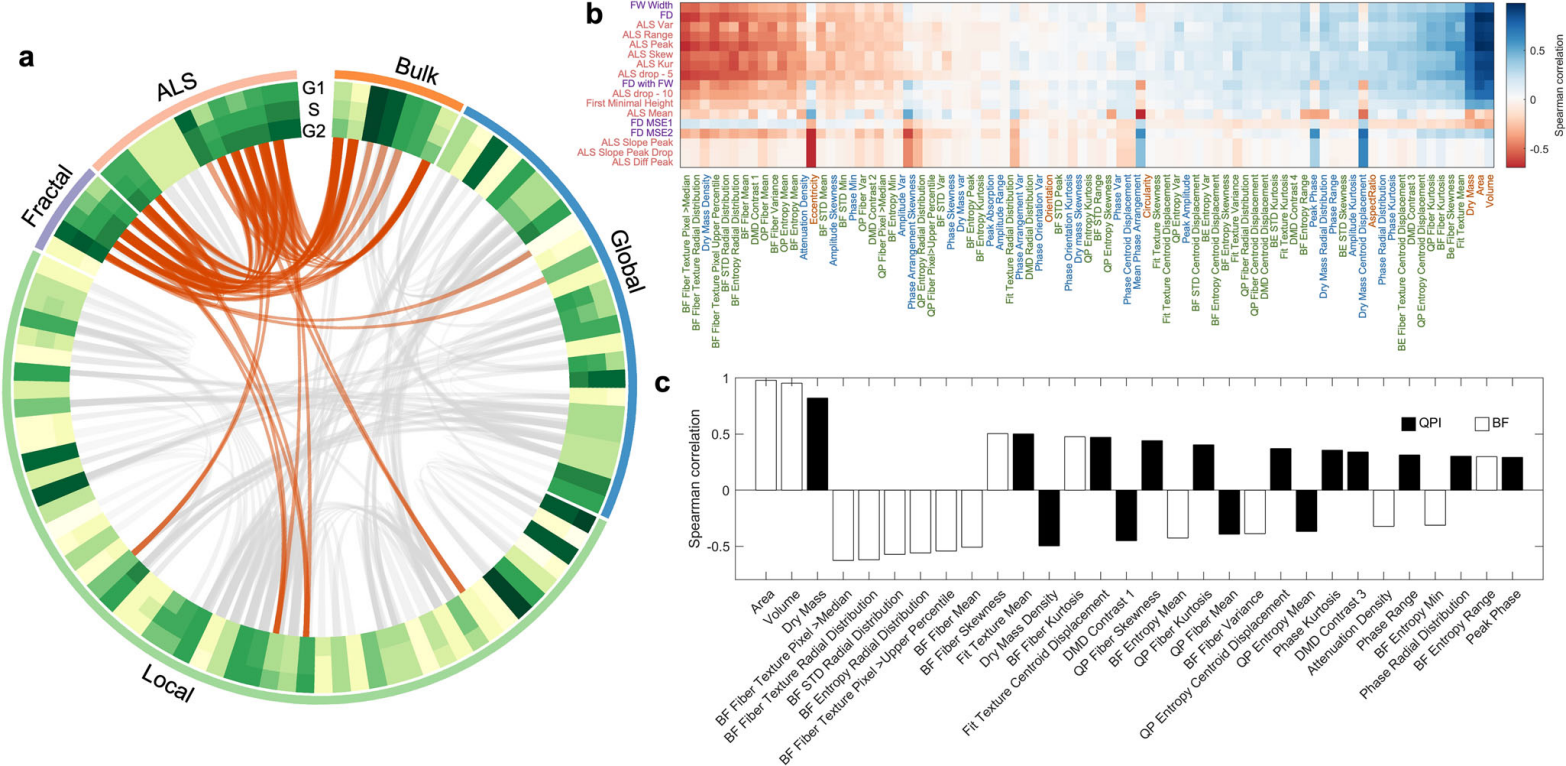
Analysis of the single-cell phenotypic correlation among the fractal, ALS, and spatial features. **a** A Circular plot summarizing the mean heatmap and the correlations among all features in cell-cycle progression. The feature type is labeled by the outmost colored ring (bulk–orange, global–blue, local–green, fractal–purple, and ALS–pink), and the full labels can be referred to Supplementary Fig. S6. The mean feature values of in different cell cycle phases are color-coded in the three ring-shaped heatmap. In the inner circle, all the feature pair with an absolute value of Spearman correlation coefficient over 0.6 are linked together by gray lines, while the Fourier-morphology connections are colored with orange specifically. Thickness of the lines is also encoded by the absolute value of correlation coefficient. **b** Spearman correlation heatmap between Fourier-domain and spatial-domain features. Fourier features are ranked by AUROC test. Spatial-domain features are ranked by the average of correlation coefficient with all Fourier features. The feature labels are colored according to the feature type specified in **a**. **c** Correlation coefficient bar chart of FD ranked by absolute value. Only 30 features with the largest coefficient magnitude with FD are listed.

Notably, the high rank of FW width could suggest that an irregular cell mass growth and distribution occur across a longer length scale (i.e., the scale-invariant property of fractals). We further visualized the expression pattern of a total of 101 morphological features (see the circular heatmap in Fig. 5a) and observed that more than half of the spatial features extracted by Euclidean geometry (from the bulk, global to local spatial features) do not show as clear changes as many ALS and fractal features across the three cell-cycle phases. The above analyses suggest that the fractal features could offer the label-free specificity and sensitivity to track the cell-cycle progression.

We further investigated if and how the common spatial features extracted by Euclidean geometry can be correlated with the ALS and fractal features during the cell cycle progression (a total of 101 dimensions)—gaining additional insight of these classical morphological features in the context of the fractal behavior (Fig. 5a–c). We observed that the Fourier features, i.e., ALS and fractal features, are in general not strongly correlated with the (Euclidean) spatial features, except a handful of 12 spatial features (i.e., ~12% of all the features) with the absolute value of Spearman correlation coefficients >0.6, see the orange lines in Fig. 5a and the heatmap in Fig. 5b). It indicates the low redundancy between these two classes of features in describing the morphological characteristics of cells. We noted that the fractal and some ALS features are particularly correlated with the cell (dry) mass and size—all of which are the important features sensitive to the cell cycle progression, consistent with the earlier feature ranking analysis (Fig. 4i). We further identified that the Fourier features are more favorably linked to the local textures (derived from both the BF and QPI images) (Fig. 5b). For instance, by ranking the spatial features according to their correlations with FD (Fig. 5c), we further identified a tight connection between the changes in FD and dry mass, the local textures that are related to the statistics of the fiber textures and structural entropy. Based on this analysis, we thus could enrich the label-free morphological profile that characterizes the biophysics of cell cycle progression. Not only the well-known features of cell and mass varies during the cell cycle, but also the local textures linked to subcellular mass density (from QPI) and optical density (from BF) distributions, many of which follow the fractal behavior. A full view of the correlation analysis can be referred to Supplementary Fig. S5.

## Discussion

Fractal characteristics of cell morphology has been well acknowledged for four decades^53^, and has also been proven indicative of complex cellular functions, especially disease progression. However, practices of defining cellular fractal phenotypes have not been widely adopted in cytometry and cell-based assay. This gap stems from that the fractal feature, e.g., FD, is a statistical measure reflecting the morphological structural complexity (especially the self-similar properties) of cellular/subcellular components. Yet, current imaging cell-based assays lack the throughput to demonstrate the fractal analysis with sufficient statistical power for delineating cellular heterogeneity, especially at the single-cell precision. In this study, we demonstrated a high-throughput morphological profiling strategy (~10,000 cells/sec) that empowers biophysical single-cell fractometry based on a catalog of label-free single-cell fractal-related features extracted from an ultrafast QPI flow cytometry platform. Specifically, the information content of the single-cell fractal profile is enriched by (1) harnessing the fractal signature of a cell is intimately linked to its light scattering characteristics, which can readily be read out by our QPI platform through *single-cell* FTLS analysis; and (2) quantifying the statistics of these single-cell fractal features, thanks to the throughput offered by the QPI platform in this work. Hence, in addition to the conventional morphological profiling strategies, single-cell biophysical fractometry offers a new dimension for parametrizing and thus fingerprinting cell morphology.

We stress that QPI, in contrast to other optical imaging modalities, has a unique capability of quantifying dry mass density distribution of a cell at high sensitivity, that is derived from the optical path length profile given by QPI (Methods)^54^. For instance, in the current multi-ATOM platform, the detection sensitivity of the optical path length was reported to be as small as 4—8 nm^25^, which corresponds to the dry mass surface density resolution of ~0.02–0.04 pg mm^−2^, taking the refractive increment of 0.19 ml g^−1^ for biological cells^55^. Therefore, biophysical single-cell fractometry described in this work measures not only the fractal properties of the cellular morphology with subcellular resolution, but also the subtle variation in the *(dry) mass* fractal characteristics.

We demonstrated that the label-free single-cell fractal profiles exhibited both biophysical implications and discriminative power for the unbiased characterization of the histologically different lung cancer subtypes and drug response effect of lung cancer cell line, and are highly correlated with the cell-cycle progression. More importantly, the fractal profile could also be integrated with the conventional morphological profile based on the spatial features extracted using the Euclidean geometry. This enables extensive feature extraction which has a two-fold benefit: first, it further augments the profiling dimensionality (and thus potentially better encode relevant biological information). This allows us to identify feature correlation, based on which further feature selection (e.g., removing noisy or redundant features^56^) can be done for extracting/refining relevant information for downstream analysis. Second, it permits us to mine the potential correlative patterns between fractal features and other spatial Euclidean features. Hence, we could gain better interpretability, and thus biophysical insights of the morphological features, in the context of fractal behavior (e.g., the correlation between FD and the local fiber textures and structural entropy identified in the cell cycle study) (Fig. 5c). This correlation analysis can also be extended to other experiments, including the lung cancer subtype identification and drug response analysis (Supplementary Fig. S7 and S8). These analyses generally show the strong relationship between fractal features and the features associated with dry mass and local textures. With this combined label-free phenotypic catalog retrieved by multi-ATOM, the morphological information embedded in both the spatial domain and Fourier domain can be both analyzed simultaneously in a comprehensive manner. We anticipate that this morphological profiling approach could enable our further understanding in how the light scattering properties are coupled with the morphological signatures for single-cell heterogeneity characterization.

We note that the fractometry strategy presented in this work could also be immediately applicable to the existing QPI modalities, especially because the imaging speed and throughput of the existing QPI modalities continue to increase to the scale comparable to those of flow cytometry^57^. As QPI can generally be adaptable with the typical fluorescence microscopes^29,58^, we anticipate that single-cell fractometry could readily be incorporated into the current fluorescence-based morphological profiling strategies which are increasingly promising in many applications, from drug discovery to basic biology research^59–61^. Using this multimodal imaging approach (especially in a high-throughput configuration^62^), future studies could aim to systematically investigate how the biophysical fractal behaviors (i.e., mass fractal studied in this work) of different subcellular organelles (e.g. nucleus^12^, mitochondria^18^, and cytoskeleton^19^) are influenced by chemical and even genetic perturbations. As the state-of-the-art single-cell computational tools become increasingly versatile in analyzing not only traditional omics data (e.g., genomics, epigenetic, transcriptomic, and proteomic etc.) but also cell morphological data^63^, we anticipate that the inclusion of fractometry in morphological profiling could facilitate the discovery of the connections between multi-omics and cell fractal at single-cell resolution morphology, (especially how the molecular signatures dictates the disease-related fractal behaviors)—thus offering a new dimension for deciphering complex cellular heterogeneity.

## Methods

### Cell culture

Seven cancer cell lines were authenticated via the Human STR profiling cell authentication service, including three adenocarcinoma cell lines (ADC, H358 (EGFR WT), HCC827 (EGFR exon 19 del), and H1975 (L858R and T790M)), two squamous cell carcinoma cell lines (SCC, H520, and H2170) and two small cell lung cancer cell lines (SCLC, H526, and H69). The breast cancer cell line used for the cell cycle experiments was MDA-MB231. Leukemia cell lines used in this work were THP-1 (TIB- 202TM) and Kasumi-1/ACC220 (CRL-2724TM). All cell lines used in this study were purchased from American Type Culture Collection (ATCC). Leukemia and lung cancer cell lines were cultured in the tissue culture flasks (surface area of 75 cm^2^) (TPP) and MB231 in100mm culture dish (Labserv), which were both placed in a CO_2_ incubator with 5% CO_2_ under 37 °C. The full culture medium was ATCC modified RPMI-1640 (Gibco) supplemented with 10% fetal bovine serum (FBS) (Gibco) and 1% antibiotic–antimycotic (Gibco). Depending on cell confluency observed by a standard light microscope, passage or medium replacement was performed 2–3 times each week.

### Drug treatment

Both Docetaxel and Gemcitabine (Abcam) were dissolved to 1 mM stock solution with DMSO under sonification and warming at 37 °C. The stock solution was then split into 1 mL aliquots and kept at −20 °C for later use within one month. An ADC cell line, H2170, was seeded in six-well plates at a seeding density of 10^5^ cells/well, and cultured in a medium with ATCC-modified RPMI-1640 (Gibco), 10% FBS (Gibco) and 1% antibiotic–antimycotic (Gibco). After 24-hour incubation, the medium in various wells were replaced with fresh medium containing targeted concentrations of drugs (IC_50_) (0 for control, 3.76 nM for Docetaxel and 7.51 nM for Gemcitabine), which was determined with reference to the online database “The Genomics of Drug Sensitivity in Cancer”^64^. The cells were then incubated at 37 °C, 5% CO_2_ for 24-hour and harvested afterwards for multi-ATOM imaging flow cytometry.

### Fluorescence labeling for cell-cycle tracking

Click-iT Plus EdU Flow Cytometry Assay Kits Alexa Fluor 488 and FxCycle PI/RNase Staining Solution were obtained from Invitrogen to define the ground truth of the cell cycle stages (G1, S, and G2 phases). The MDA-MB231 cell culture was firstly renewed for 8 mL medium mixed with 8 μL of 10 mM EdU staining solution. After 2-hour incubation, the cells were harvested by 0.25% Trypsin (Thermo Scientific) and washed by PBS with 1% BSA. The cells were then brought to protection from light for the following steps. The centrifuged cells were fixed by 100 μL Click-iT fixative (4% paraformaldehyde in PBS) and then permeabilized by the permeabilization and wash reagent (sodium azide) with 15-minute incubation. Next, 500 μL Click-iT Plus reaction cocktail was added into 100 μL cell suspension for 30 min under room temperature. After washing with 3 mL Click-iT permeabilization and wash reagent, the cell pellet was then mixed with 500 μL Click-iT permeabilization and wash reagent and 500 μL FxCycle PI/RNase staining solution for 30 min at room temperature. Lastly, after washing away the staining solution, PBS was added to make up a total volume of 7 mL cell suspension for the subsequent imaging experiments.

### Microfluidic channel fabrication

The channel was designed for optimization of inertial focusing to generate an in-focus single-cell stream under fast microfluidic flow (>2 m/s) and then fabricated by polydimethylsiloxane (PDMS) by soft lithography technique. Firstly, a layer of photoresist (SU-82025, MicroChem, US) was coated on a silicon wafer by a spin coater (spinNXG-P1, Apex Instruments Co., India), which was soft-baked two times (65 °C for 3 min and 95 °C for 6 min). After cooling down under room temperature, a computer-aided design (CAD) pattern was transferred onto the photoresist by a maskless soft lithography machine (SF-100 XCEL, Intelligent Micro Patterning, LLC, US) through a 4-second exposure and a two-step post-baking (65 °C for 1 min and 95 °C for 6 min). After photoresist development with SU-8 developer (MicroChem, US) for 5 min, the wafer was rinsed and dried for subsequent PDMS mixture pouring, which was mixed with PDMS precursor (SYLGARD^®^ 184 Silicone Elastomer kit, Dow Corning, US) and curing agent at a ratio of 10:1. The height control of the imaging section in the microfluidic chip was performed by placing a custom-designed acrylic block on the wafer, yielding a channel dimension of 30 μm in height and 60 μm in width. After the channel curing in an oven at 65 °C for 2 h before demolding, a biopsy punch (Miltex 33-31 AA, Integra LifeSciences, US) was used to punch two holes for later tube insertion as the inlet and outlet of the channel. Afterwards, the bonding between the channel and glass slide was activated by oxygen plasma (PDC-002, Harrick Plasma, US) and oven baking under 65 °C for 30 min. Lastly, plastic tubings were inserted into the chip as channel inlet/outlet (BB31695-PE/2, Scientific Commodities, Inc., US).

### Multi-ATOM imaging

Multi-ATOM combines the time-stretch imaging technique^65,66^ and phase gradient multiplexing method to retrieve complex optical field information (including the bright-field and quantitative-phase contrasts) of the cells at high speed in an interferometry-free manner (Fig. 1a). Detailed working principle and experimental configuration were reported previously^8,25,26^. In brief, a wavelength-swept laser source was firstly generated by a home-built all-normal dispersion (ANDi) laser (centered wavelength: 1064 nm; bandwidth: ~10 nm; repetition rate: 11 MHz; pulse width = ~12 ps). The laser pulses were temporally stretched in a single-mode dispersive fiber (group-velocity dispersion (GVD): 1.78 ns/nm), and were then amplified by an ytterbium-doped fiber amplifier module (output power = 36 dBm with an on–off power gain = 36 dB). The pulsed beam was subsequently launched to and spatially dispersed by a diffraction grating (groove density = 1200/mm) into a 1D line-scan beam which was projected orthogonally onto the cells flowing in the microfluidic channel. This line-scan beam was transformed back to a single collimated beam after passing through a double-pass configuration formed by a pair of objective lenses (N.A. = 0.75/0.8). Afterwards, the beam conveying phase-gradient information of the cell was split into 4 replicas by a one-to-four de-multiplexer, where each beam profile (${{I}_{x}}^{+}$, ${{I}_{x}}^{-},{{I}_{y}}^{+}$, ${{I}_{y}}^{-}$) was half-blocked by a knife edge from four different orientations (left, right, top, and bottom) respectively. Recombining the 4 beams by a four-to-one fiber-based time-multiplexer, we were able to detect the line-scan phase-gradient information in 4 directions in time sequence at high speed by a single-pixel photodetector (electrical bandwidth = 12 GHz (Newport, US)). The digitized data stream was processed by a real-time field programmable gate array (FPGA) based signal processing system (electrical bandwidth = 2 GHz, sampling rate = 4 GSa/s) for primary cell detection and image segmentation with a processing throughput of >10,000 cells/s in real-time. These segmented phase-gradient images of cells were sent to four data storage nodes (memory capacity >800 GB) through four 10 G Ethernet links, which were reconstructed to 2D complex-field information following a complex Fourier integration algorithm, detailed elsewhere^25^ (Fig. 1b, c).

### ALS validation using microbeads

The calibration experiment was conducted by multi-ATOM using polymethyl methacrylate (PMMA) microbeads as a standard sample, which have homogenous refractive index and normative spherical shape. Microbeads were pipetted out from the stock solution and then centrifuged at 1000 rpm for 5 mins. After removing the supernatant, microbeads were resuspended in 10 mL of 5% BSA solution, followed with 10-min incubation with sonication at room temperature to saturate any surface coating and avoid unwanted aggregation. The microbeads were then resuspended in PBS with desired concentration after centrifugation (1000 rpm, 5 mins). After flowing the bead sample into multi-ATOM imaging platform, we obtained their FTLS patterns by Fourier transform, and obtained the theoretical simulation results^8^ and Mie scattering prediction of ball-shape phase objects with the same radius of bead size (Fig. 1d).

### Fluorescence detection

2-channel (i.e., 2-color) fluorescence detection was also synchronized with multi-ATOM, i.e., the bright-field, quantitative-phase contrasts and fluorescence signal from the same cell can be detected simultaneously. It was employed to generate the ground truth of the cell-cycle stages for single-cell fractometry based on multi-ATOM images. When flowing through the imaging section in the microfluidic channel, the cells were excited by two continuous wave (CW) lasers (wavelength: 488 nm and 532 nm) simultaneously to generate epi-fluorescence signals, which were detected by two photomultiplier tubes (PMT) in the end. Frequency modulation was done (11.8 MHz and 35.4 MHz respectively) by a multichannel direction digital synthesizer to multiplex the PMT-received signals. After digital demodulation and low-pass filtering, the two fluorescence signals of each segmented cell were synchronized with multi-ATOM signal by the same FPGA configuration.

### Single-cell fractal analysis

The complex field at the image plane of the flowing cell measured by multi-ATOM is denoted as

1 $$E\left(x,y\right)=A\left(x,y\right){e}^{j\phi \left(x,y\right)}$$

where $A\left(x,y\right)$ is amplitude profile and $\phi \left(x,y\right)$ is the quantitative phase profile. In multi-ATOM, the phase gradient along the x-direction $\frac{\partial \phi }{\partial x}$ is first extracted based on the two raw knife-edged images (cut from left and right orientations): ${{I}_{x}}^{+}\left(x,y\right)$ and ${{I}_{x}}^{-}\left(x,y\right)$, through the relationship:

2 $$\frac{\partial \phi }{\partial {x}_{s}}\propto \frac{{I}_{x}^{+}-{I}_{x}^{-}}{{I}_{x}^{+}+{I}_{x}^{-}}$$

the same expression of the phase gradient along the y-direction $\frac{\partial \phi }{\partial y}$ can be applied to the knife edges in the y-direction, ${{I}_{y}}^{+}\left(x,y\right)$ and ${{I}_{y}}^{-}\left(x,y\right)$. Hence, the quantitative phase $\phi \left(x,y\right)$ was then obtained by applying complex Fourier integration on the phase gradient images captured in multi-ATOM$\,\nabla \phi (x,y)=\frac{\partial \phi }{\partial {x}_{s}}(x,y)+i\frac{\partial \phi }{\partial {y}_{s}}(x,y)$:

3 $$\phi \left(x,y\right)={CF} \,\cdot \, {Im}\left\{{{{{{{\mathcal{F}}}}}}}^{-1}\left\{{NF}\,{{{{{\mathscr{\cdot }}}}}} \, {{{{{\mathcal{F}}}}}}\left[\nabla \phi \left(x,y\right)\right]\right\}\right\}$$

$${where}\,{NF}=\left\{\begin{array}{cc}\frac{{FOV}}{\left[2\pi j \, \cdot \, k\left(x,y\right)\right]}, & k\left(x,y\right)\,\ne \, 0\\ 0, \hfill& k\left(x,y\right)=0\end{array}\right.$$

where ${Im}$ is the imaginary part of a complex number; and ${{{{{{\mathscr{F}}}}}}}^{-1}$ is inverse Fourier transform operator; ${NF}$ is a normalization factor for quantifying the phase and avoiding singularity in the integration operation; $k\left(x,y\right)$ is the 2D wavenumber; ${FOV}$ is the 2D field-of-view; ${CF}$ is the calibration factor for correcting the systematic phase deviation arise from non-ideal system setting^8^. On the other hand, the amplitude image of the cell ($A(x,y)$) is the sum of two images obtained from opposite knife edges normalized by the background (i.e., $B$, regions without samples).

4 $$A\left(x,y\right)=\frac{1}{B}\left({I}_{x}^{+}+{I}_{x}^{-}\right)=\frac{1}{B}\left({I}_{y}^{+}+{I}_{y}^{-}\right).$$

Subsequently, the complex field at the image plane is then numerically propagated to the far field using the Fourier transform operation—effectively yielding the (far-field) scattered light-field pattern $S\left({k}_{x},{k}_{y}\right)=\,{{{{{\mathcal{F}}}}}}[E\left(x,y\right)]$^27^, from which the fractal properties of cell can be measured. We then convert the scattered light pattern into an angular light scattering (ALS) profile $S\left(q\right)$ in which scattered light intensity is averaged over rings of constant wave vector $q=4\pi /\lambda {{\sin }}({{{{{\rm{\theta }}}}}}/2)$, where $\theta$ is the *polar* scattering angle^27^.

To quantify the fractal characteristics from the ALS, we also define a density-density correlation function in the real space, which is related to the ALS intensity via the Fourier transform relationship as the refractive index variation arises from density fluctuation $\rho (r)$^40^:

5 $${{{{{\mathcal{F}}}}}}\left[{\left|S\left(\vec{{{{{{\boldsymbol{\theta }}}}}}}\right)\right|}^{2}\right]=\left\langle S\left(\vec{{{{{{\bf{r}}}}}}}^{\,\prime} \right){S}^{* }\left(\vec{{{{{{\bf{r}}}}}}}^{\,\prime} +r\hat{\theta }\right)\right\rangle \propto \left\langle \rho \left(\vec{{{{{{\bf{r}}}}}}}^{\,\prime} \right){\rho }^{* }\left(\vec{{{{{{\bf{r}}}}}}}^{\,\prime} +r\hat{\theta }\right)\right\rangle \equiv {C}_{\rho }(r)$$

where $\rho (r)$ means the density at point *r*, and thus *C*_*ρ*_(*r*) is the density correlation of an arbitary pair of occupied particles with a correlation distance of *r*^67^. As It is known that the mass distribution of a fractal object can be expressed as^9^, *m*(*r*) ∝ *r*^*FD*^, where *m*(*r*) is the mass within a sphere of radius *r*. It can be also linked to *C*_*ρ*_(*r*) as^67^:

6 $$m\left(r^{\prime} \right)\propto \,{\int }_{0}^{r^{\prime} }{C}_{\rho }(r){d}^{3}r$$

which indicates ${C}_{\rho }\left(r\right)\propto {r}^{{FD}-3}$. Hence, the Fourier transform of an ALS profile will obey an inverse power law relationship, i.e., ${{{{{\mathcal{F}}}}}}\left[{\left|S\left(\theta \right)\right|}^{2}\right]\propto {r}^{-\alpha }$, where $\alpha$ is the exponent, and FD can be expressed as:

7 $${FD}=3-\alpha$$

Therefore, by fitting the slope $\alpha$ of the log-scaled plot of ${{{{{\mathcal{F}}}}}}\left[{\left|S\left(\vec{\theta }\right)\right|}^{2}\right]$ versus the correlation distance $r$ we could quantify the FD, i.e., $3-\alpha$ (Fig. 1e). Besides, FW is defined as the interval of correlation distance with the most prominent downwards slope in ${C}_{\rho }(r)$ (See the equation stated in Supplementary Table S1). Here the criteria of the FW definition include: (1) the fractal analysis should fall well within the image resolution achieved in our system; (2) the short and long correlation distance segments are excluded. These are the regions where the downwards slope in the correlation curve is smaller than the slope of overall fitting—which indicating that the linearity is not significant. We further used “multiple segments” of FW to extract the FD value in this work, including both overall fitting (FD) and fitting within fractal window (FD with FW), in order to more comprehensively characterize the cellular fractality in different ways (Supplementary Table S1).

### Statistics and reproducibility

Feature extraction and downstream statistical analysis was both performed with MATLAB (MathWorks, USA). Large sample size was obtained to guarantee reproducibility, which was 38,001 for cancer cell line subtype identification, 10,773 for drug response analysis, and 15,884 for cell cycle tracking, respectively. The statistical significance of typical features was visualized by violin plot^68^ and quantified by effect size^47^. The data distribution of feature profile was visualized by UMAP^48^ and quantified by ROC curve.

### Reporting summary

Further information on research design is available in the Nature Portfolio Reporting Summary linked to this article.

## Supplementary information

Supplementary Information

Reporting Summary

## Data Availability

A test dataset used in this paper can be accessed on the following link for your reference: 10.5281/zenodo.7787607. Due to the large size of the raw image data, the complete data supporting the findings of this study are available from the corresponding author upon reasonable request.

## Code availability

Customized MATLAB code relevant to this paper can be accessed on the following link for your reference: 10.5281/zenodo.7787607.
