# Supplementary material for: Morphological profiling by high-throughput single-cell biophysical fractometry
Source: Commun Biol. 2023 Apr 24;6:449. doi: 10.1038/s42003-023-04839-6 (PMC10126163; doi:10.1038/s42003-023-04839-6)

Supplementary information

Morphological profiling with high-throughput single-cell biophysical fractometry

Ziqi Zhang[1], Kelvin C. M. Lee[1], Dickson M. D. Siu[1], Michelle C.K. Lo[1], Queenie T. K. Lai[1], Edmund Y.M. Lam[1] and Kevin K. Tsia[1,2]

[1]Department of Electrical and Electronic Engineering, The University of Hong Kong, Pokfulam Road, Hong Kong
[2]Advanced Biomedical Instrumentation Centre, Hong Kong Science Park, Shatin, New Territories, Hong Kong
**E-mail: tsia@hku.hk*

Table of content

Supplementary Table S1: Full list of Fourier-domain features.
Supplementary Note: Definitions of spatial features
Supplementary Fig. S1: Label-free single-cell image capture of 7 lung cancer cell lines.
Supplementary Fig. S2: Statistics of typical spatial-domain features of MB231 cell line.
Supplementary Fig. S3: 2-color fluorescence detection result by standard flow cytometry for cell cycle determination.
Supplementary Fig. S4: Feature performance on identifying the three cell cycle phases of MB231 cell line in one-versus-all mode.
Supplementary Fig. S5: Correlation matrix of all phenotypes of MB231 cell line (including both Fourier and spatial domain).
Supplementary Fig. S6: Circular plot combining the mean phenotypic heatmap and correlations of MB231 cell line with the feature labels.
Supplementary Fig. S7: Circular plot combining the mean phenotypic heatmap and correlations of lung cancer cell lines with the feature labels.
Supplementary Fig. S8: Circular plot combining the mean phenotypic heatmap and correlations of drug treated H2170 cell line with the feature labels.

Table S1: Full list of Fourier-domain features. 17 dimensions in total are extracted from ALS and correlation function to characterize the single-cell heterogeneity by multi-ATOM.

Feature name	Physical meaning	Notation	Equation
FD	Fractal dimension	FD	$FD = 3 - \alpha, [\alpha, \beta] = \frac{\boldsymbol{X}^T\boldsymbol{Y}}{\boldsymbol{X}^T\boldsymbol{X}}$ where $\boldsymbol{X} = \begin{bmatrix} \log(r_1) & 1 \\ \vdots & \vdots \\ \log(r_n) & 1 \end{bmatrix}, \boldsymbol{Y} = \begin{bmatrix} \log[f(r_1)] \\ \vdots \\ \log[f(r_n)] \end{bmatrix}$, $f(r) = F[S(\theta)]^2$, n is the length of correlation function
FW Width	The width of fractal window	$r_j - r_i$	Find the interval $[r_i, r_j]$ where $\frac{\log[f(r_k)] - \log[f(r_{k-1})]}{\log(r_k) - \log(r_{k-1})} < \alpha \ (i < k \leq j)$
FD with FW	Fitted fractal dimension within the fractal window	FD'	$FD' = 3 - \alpha', [\alpha', \beta'] = \frac{\boldsymbol{X}'^T\boldsymbol{Y}'}{\boldsymbol{X}'^T\boldsymbol{X}'}$ where $\boldsymbol{X}' = \begin{bmatrix} \log(r_i) & 1 \\ \vdots & \vdots \\ \log(r_j) & 1 \end{bmatrix}, \boldsymbol{Y}' = \begin{bmatrix} \log[f(r_i)] \\ \vdots \\ \log[f(r_j)] \end{bmatrix}$
FD MSE1	Fitting error within the fractal window		$\frac{(\boldsymbol{X}'\boldsymbol{A}' - \boldsymbol{Y}')^T(\boldsymbol{X}'\boldsymbol{A}' - \boldsymbol{Y}')}{n}$ $where\ \boldsymbol{A}' = [\alpha', \beta']$
FD MSE2	Fitting error of the overall function		$\frac{(\boldsymbol{XA} - \boldsymbol{Y})^T(\boldsymbol{XA} - \boldsymbol{Y})}{n}$ $where\ \boldsymbol{A} = [\alpha, \beta]$
ALS	Angular light scattering (1D function)	$S(\theta)$	$S(\boldsymbol{q}) = \int E(\boldsymbol{r})e^{-i\boldsymbol{q}\cdot\boldsymbol{r}}d^2\boldsymbol{r} = \mathcal{F}[E(\boldsymbol{r})]$, where $\boldsymbol{r}$ stands for the spatial vector, and $\theta = 2sin^{-1}(\frac{q\lambda}{4\pi})$
ALS difference	The scattering difference of adjacent sample points (1D function)	$\Delta S(\theta)$	$S(\theta) - S(\theta - \Delta\theta)$, where $\Delta\theta$ means the angular resolution of ALS measurement
ALS slope	The point-by-point slope of scattering curve (1D function)	$d(\theta)$	$\Delta S(\theta)/\Delta\theta$
ALS Drop – 5°	The intensity difference of scattering function from 0° to 5°		$S(0°) - S(5°)$

ALS Drop – 10°	The intensity difference of scattering function from 0° to 10°		$S(0°) - S(10°)$
First Minimal Height	The intensity difference of from 0° to first local minimal		$S(\theta_0) - S(0°)$, where θ_0 is the minimal value of θ to make $\frac{\mathrm{d}}{\mathrm{d}\theta}\mathrm{S}(\theta_0) = 0$ and $S(\theta_0) \leq S(\theta_0 \pm \Delta\theta)$
ALS Diff Peak	Maximum scattering difference		$\max\,[\Delta S(\theta)]$
ALS Slope Peak Drop	The scattering difference at the angle with the maximum slope		$\max[d(\theta)] \cdot \Delta\theta$
ALS Slope Peak	Maximum point-by-point slope		$\max\,[d(\theta)]$
ALS Mean	Mean of scattering function	$\bar{S}$	$\mathrm{mean}[S(\theta)]$
ALS Var	Variance of scattering function	σ_S^2	$\sum_\theta[S(\theta) - \bar{S}]^2\,/(N-1)$, where N is the length of ALS
ALS Skew	Skewness of scattering function		$\frac{\sum_\theta[S(\theta) - \bar{S}]^3}{N \cdot \sigma_S^3}$
ALS Kur	Kurtosis of scattering function		$\frac{\sum_\theta[S(\theta) - \bar{S}]^4}{N \cdot \sigma_S^4}$
ALS Range	Intensity range of scattering function		$\max[S(\theta)] - \min\,[S(\theta)]$
ALS Peak	Maximum value of scattering function		$\max\,[S(\theta)]$

Supplementary Note: Definitions of spatial features

These features are displayed in **Supplementary Fig. S5-8** in order to show the correlations between them with fractal/ALS features. Here below are the detailed descriptions of the features:

Bulk features
Bulk features (e.g. the cell size, cell mass, and the cell shape (i.e. Circularity, Eccentricity, Aspect Ratio, Orientation) were extracted according to the mask of the cell in QPI (i.e., ϕ images), using basic thresholding.

Global texture features
They were extracted in each BF and ϕ images, based on the statistical distribution of the gray-scale values in the images. They include the basic statistical moments of the global distribution (i.e. mean, variance, skewness and kurtosis), and the peak, minimum values and the range of the distribution. Averaged dry mass density was also included. The phase arrangement features characterize the phase distribution along the radial directions. The phase orientation phenotypes describe the relationship of phase values, its angular position and angular

“repetitiveness”. The phase values were first represented in the angular coordinates. Then the distribution was Fourier transformed to obtain a distribution of phase in the angular frequency domain. The statistical moments of this distribution were used as the phenotypes. We also included the centroid displacement and radial distribution of the mass density phenotypes. Centroid displacement measures the displacement of the weighted centroid of the mass/phase from the unweighted centroid obtained from the mask alone. Radial distribution characterizes the tendency of distribution going closer to the edge or to the centre of the cell.

Local texture features

To extract the local texture features, different local kernals (filters) were employed. They include entropy filter with a kernel size of 2μm, standard deviation filter with kernel size of 1 μm, and Hessian-based multiscale filter. The features extracted with these filters have “Entropy”, “STD” for optical density features and mass density features, and “Fiber” in their feature names. Hence, Phase STD, Phase Entropy and Phase Fiber phenotypes are equivalent to BF STD, BF Entropy and BF Fiber phenotypes, but performed in the quantitative phase map of the cell. We also quantified the centroid displacement and radial distribution of these local texture features. Finally, the Fit Texture phenotypes were obtained by characterizing the statistical moments of the profile that emphasizes the high spatial frequency of the phase. It was obtained by subtracting the phase profile of the cell with a smoothed phase profile of the cell (computed from a fitted polynomial surface, along the x and y directions up to the 5th degree). The subtracted profile thus contains the high spatial frequency details of the cell.

Figure S1: Label-free single-cell image capture of 7 lung cancer cell lines. Random-selected **(a)** phase gradient image in x-direction (DICx), **(b)** quantitative phase images (QPI) and **(c)** summarizing curve of the correlation function (ALS FT) of 7 lung cancer cell lines, respectively. Scalar bar is 5 µm. Shaded area indicates the statistical variance.

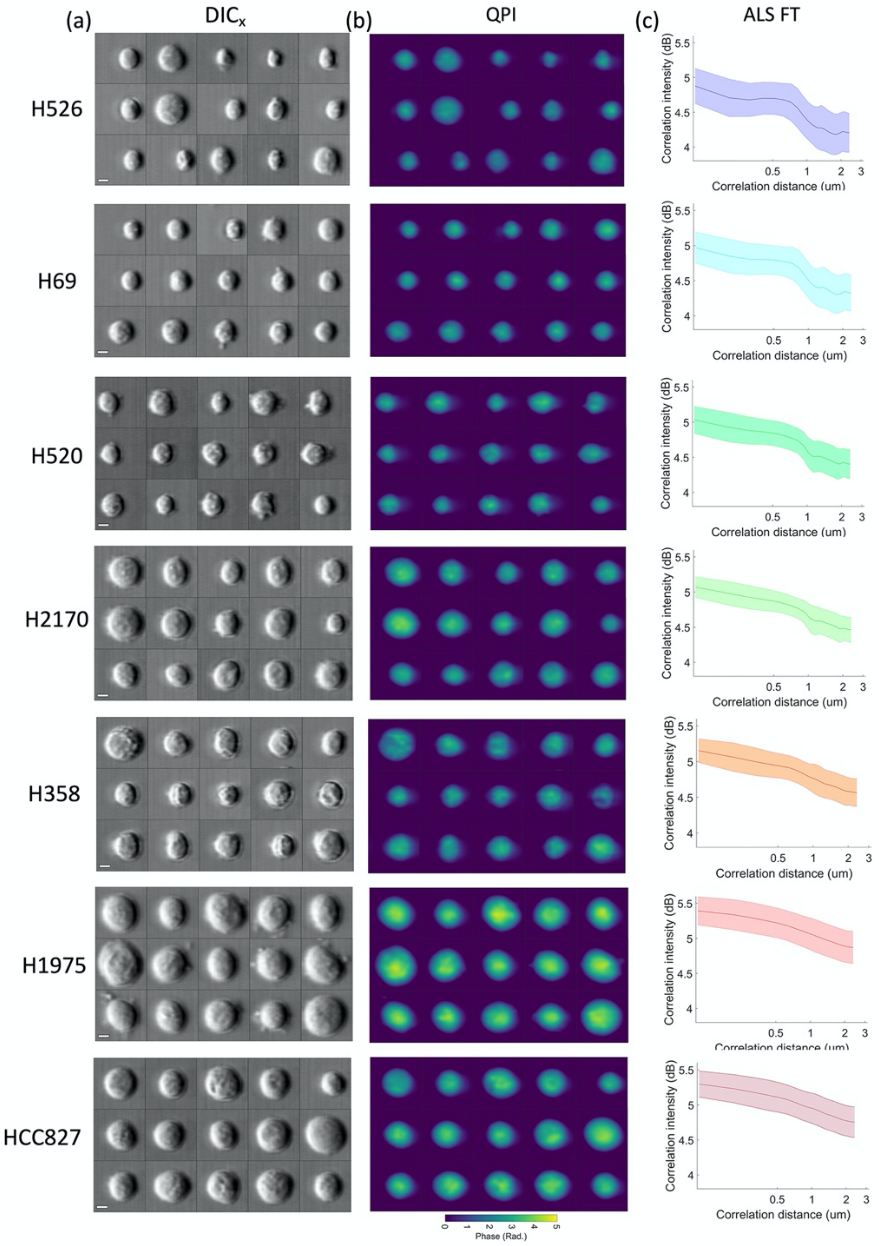

Figure S2: Statistics of typical spatial-domain features of MB231 cell line. Violin plot of **(a)** Cell size, **(b)** dry mass, and **(c)** dry mass density of cells in different cell cycle phase.

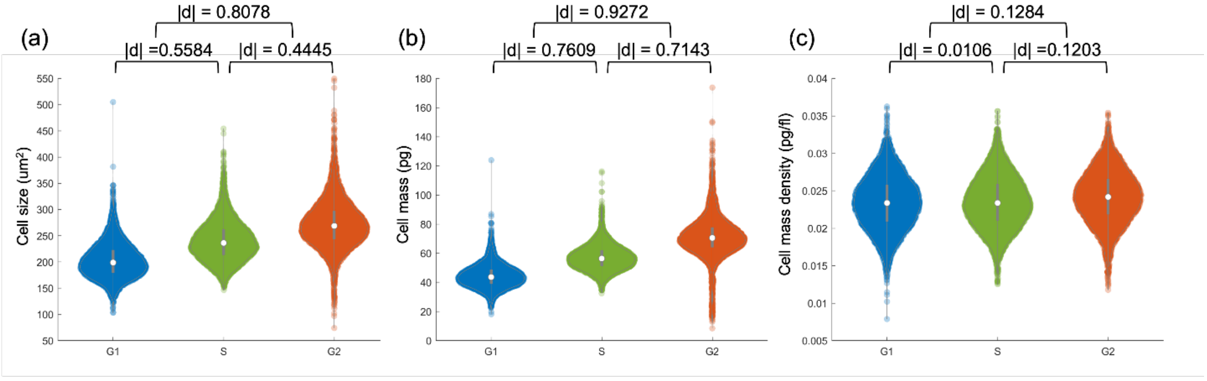

Figure S3: 2-color fluorescence detection result by standard flow cytometry for cell cycle determination. The cytometer used in this trail is *BD FACSAria™ III*. PI intensity is quantified in *PE-Texas Red* channel, whereas EdU intensity is detected in *FITC* channel. 50,910 cells are captured here in total, and 45,112 cells after gating of debris and aggregates possess a similar pattern showing cell cycle distribution with **Fig. 4b**.

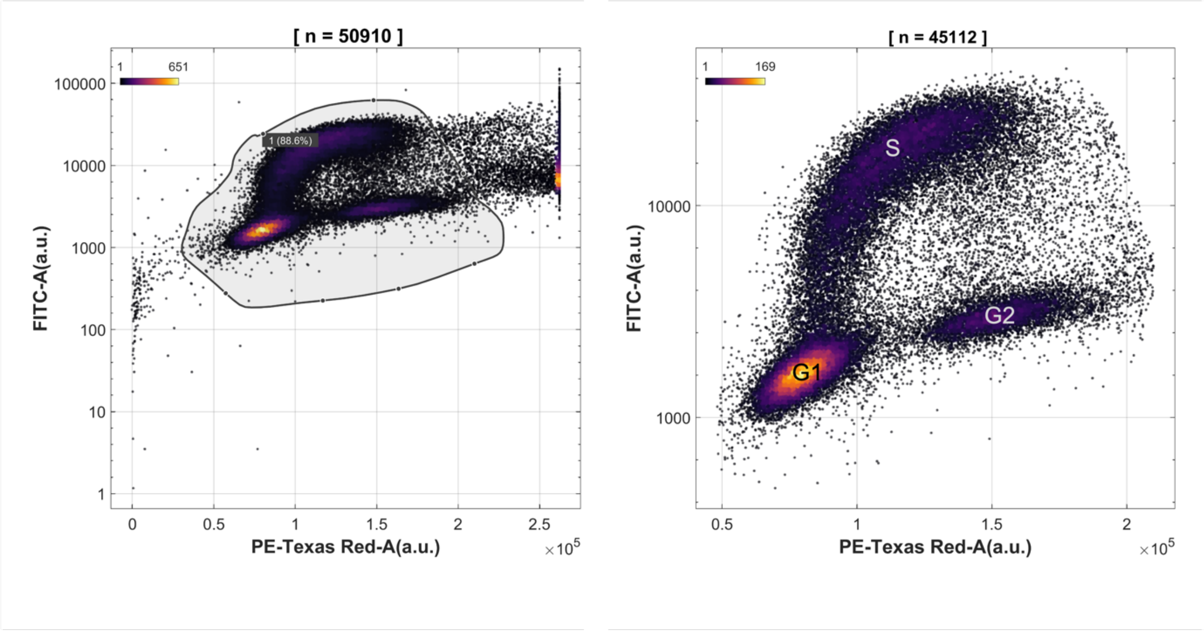

Figure S4: Feature performance on identifying the three cell cycle phases of MB231 cell line in one-versus-all mode. Features are ranked by the average area-under-curve of the receiver operating characteristics (AUROC) and only the top 15 features are shown.

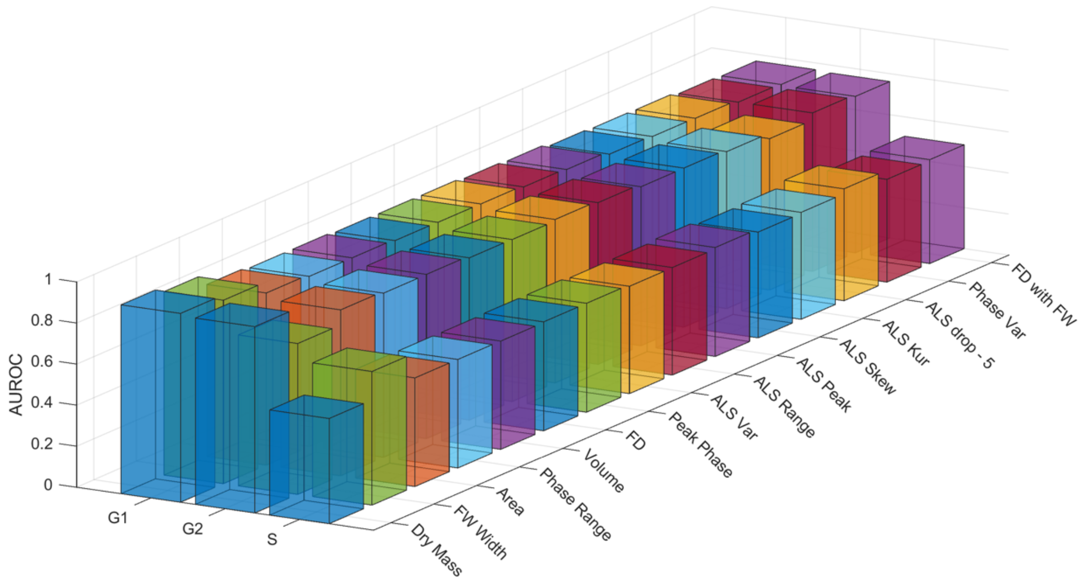

Figure S5: Correlation matrix of all phenotypes of MB231 cell line (including both Fourier and spatial domain). Blue and red color represents the positive and negative Spearman correlation coefficient, respectively, and the color level and size of the dot are both encoded with the magnitude of correlation coefficient. Features are arranged according to their feature type (i.e., bulk, global, local, fractal and ALS).

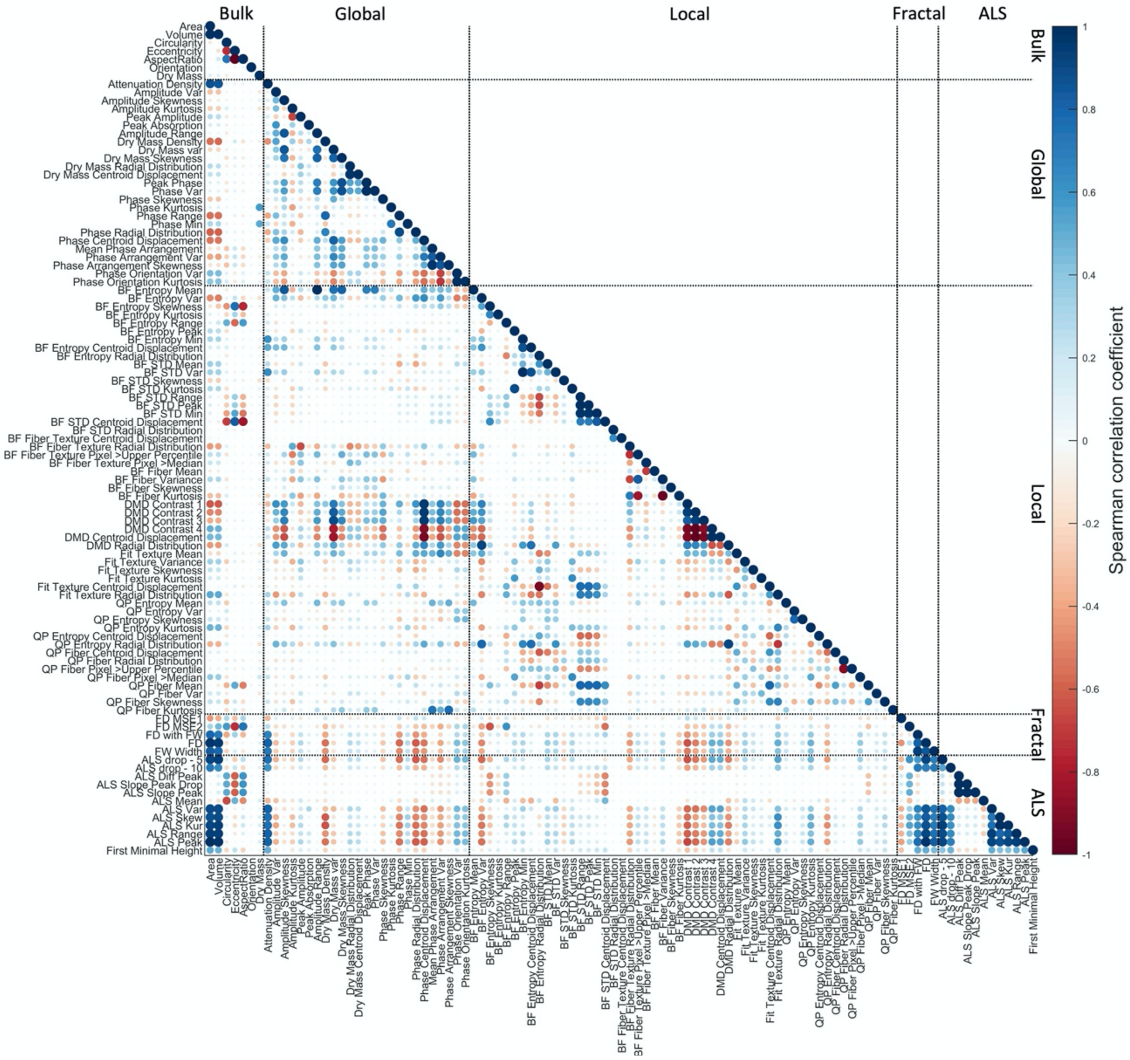

Figure S6 (same as Fig. 5a): Circular plot combining the mean phenotypic heatmap and correlations of the cell cycle analysis of the MDA-MB231 cells with the feature labels.

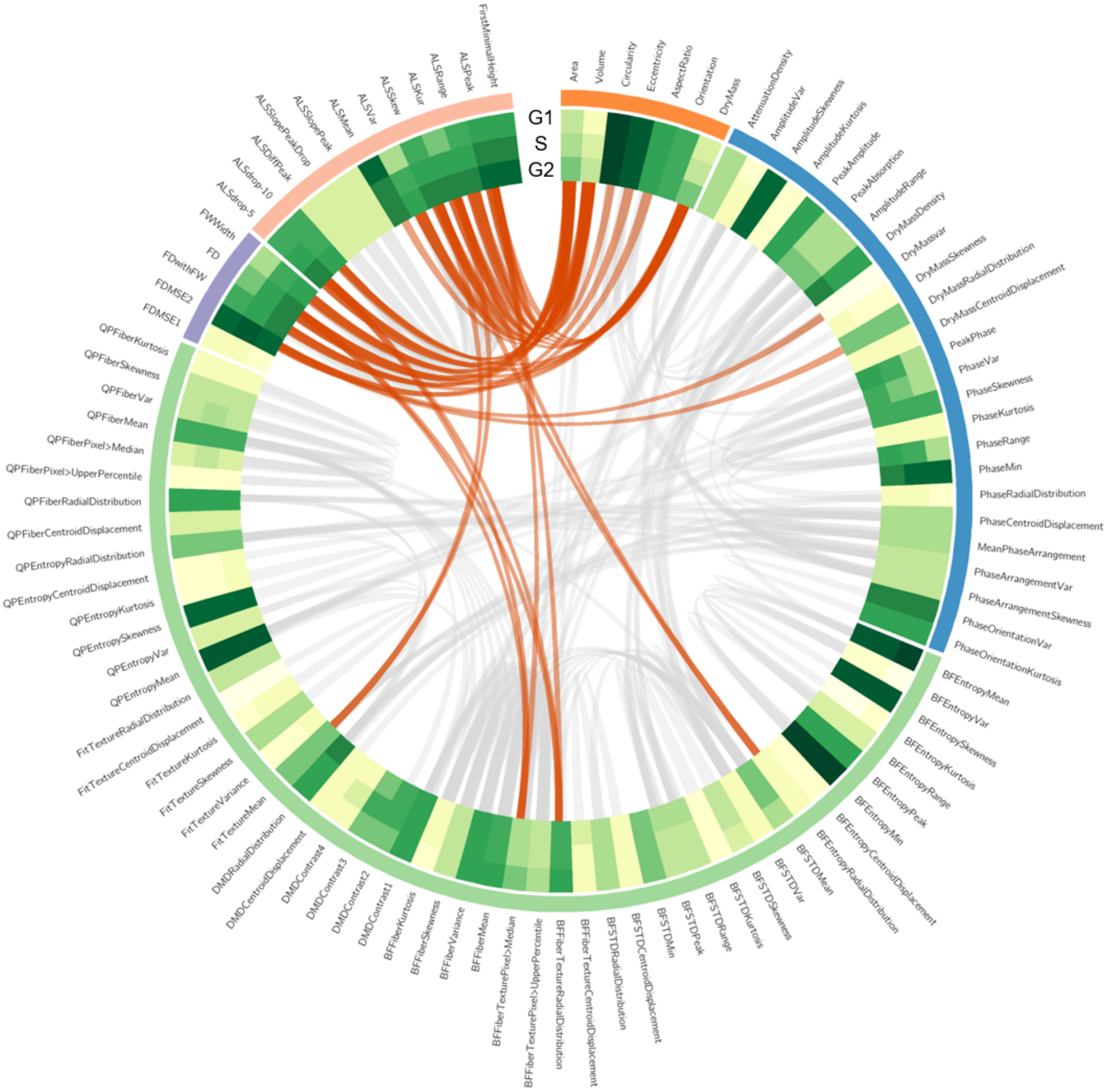

Supplementary Fig. S7: Circular plot combining the mean phenotypic heatmap and correlations of lung cancer cell lines with the feature labels. The feature type is labelled by the outmost colored ring (bulk - orange, global - blue, local - green, fractal – purple and ALS - pink). The mean feature values of in different cell cycle phases are color-coded in the three ring-shaped heatmap. In the inner circle, all the feature pair with an absolute value of Spearman correlation coefficient over 0.8 are linked together by gray lines, while the Fourier-morphology connections are colored with orange specifically. Thickness of the lines is also encoded by the absolute value of correlation coefficient.

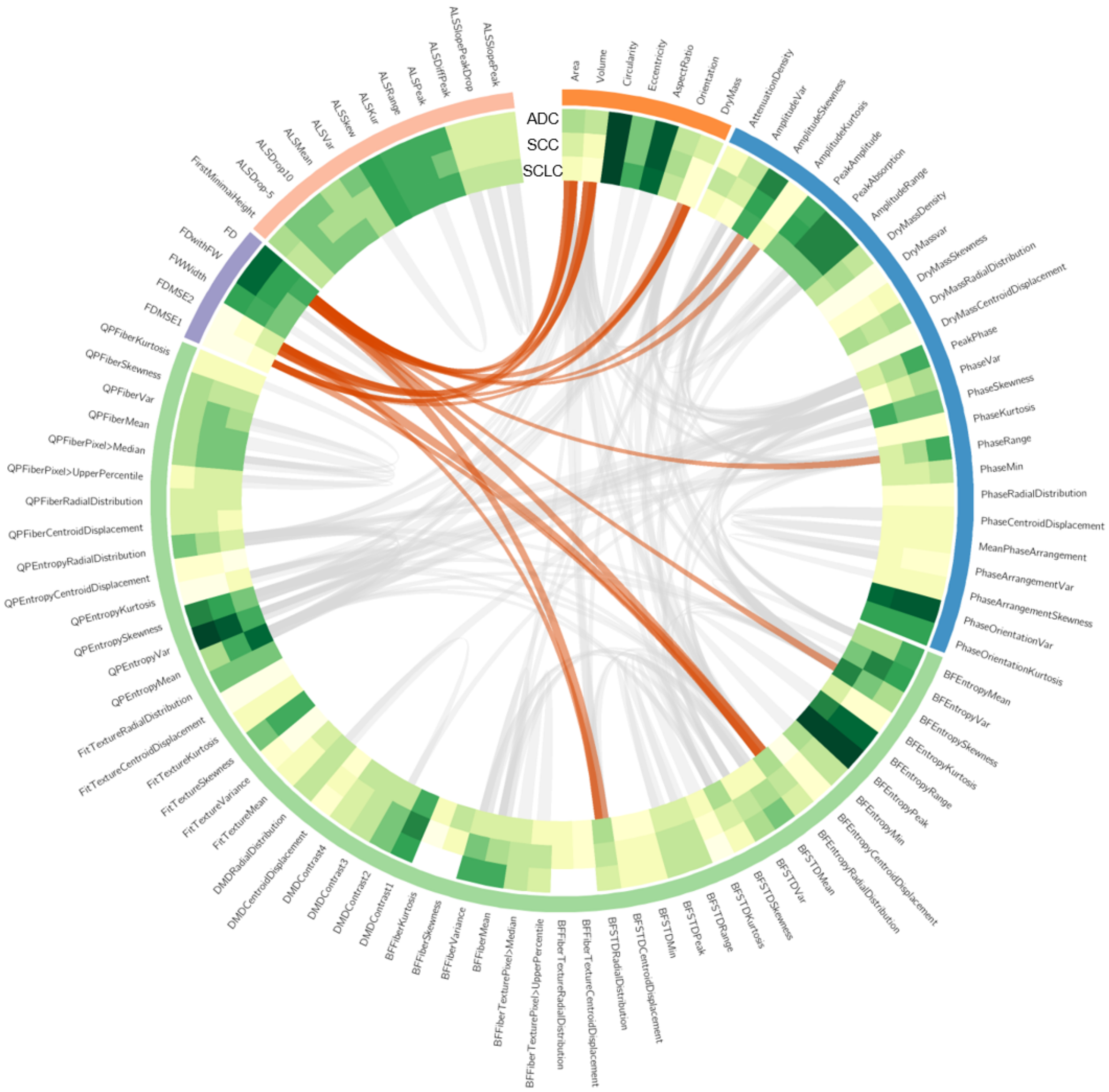

Supplementary Fig. S8: Circular plot combining the mean phenotypic heatmap and correlations of drug treated H2170 cell line with the feature labels. The feature type is labelled by the outmost colored ring (bulk - orange, global - blue, local - green, fractal – purple and ALS - pink). The mean feature values of in different cell cycle phases are color-coded in the three ring-shaped heatmap. In the inner circle, all the feature pair with an absolute value of Spearman correlation coefficient over 0.7 are linked together by gray lines, while the Fourier-morphology connections are colored with orange specifically. Thickness of the lines is also encoded by the absolute value of correlation coefficient.

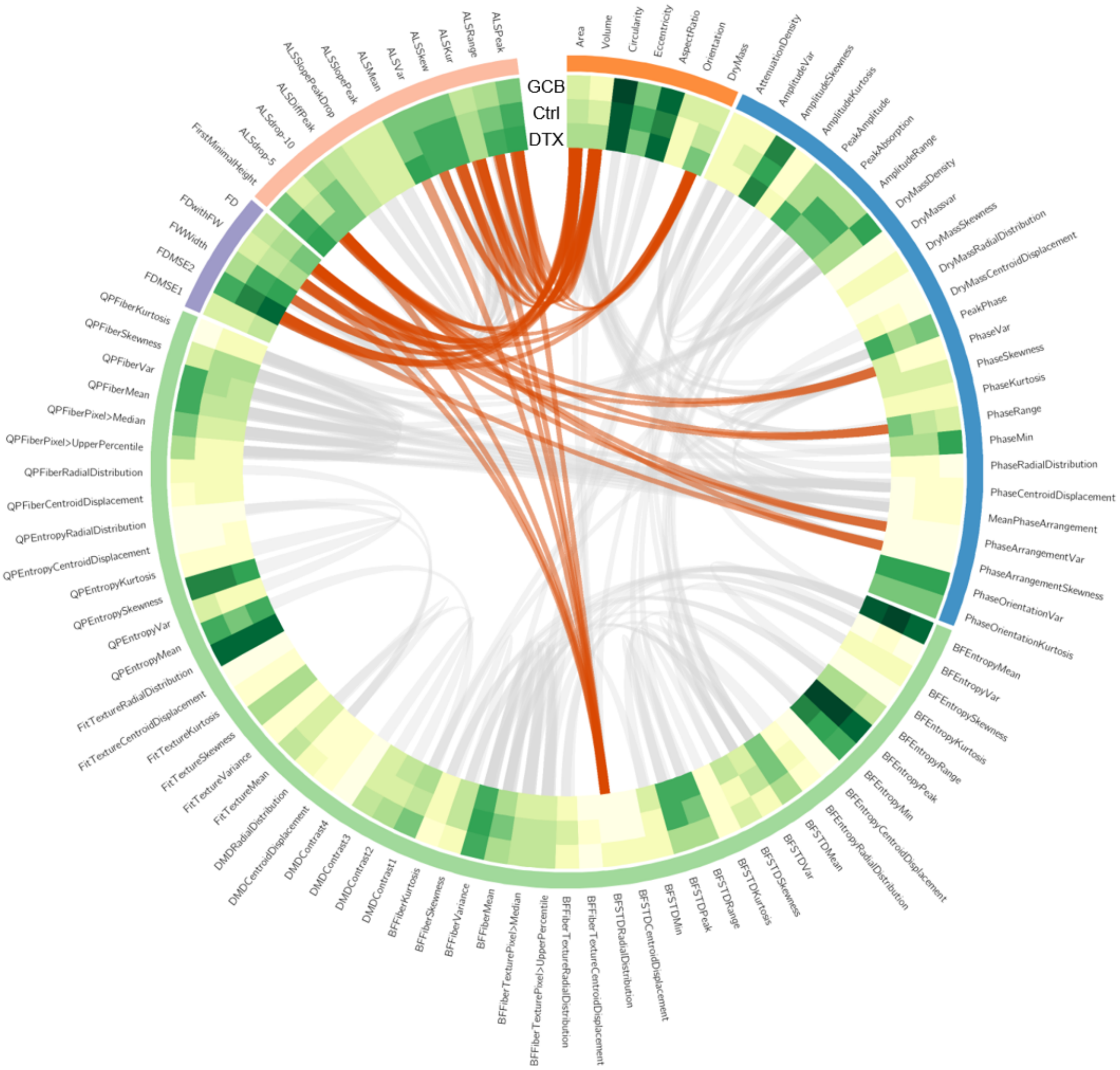

Supplement: Supplementary file 1 — Supplementary Information [file 42003_2023_4839_MOESM1_ESM.pdf]
