# Supplementary material for: Morphological profiling by high-throughput single-cell biophysical fractometry
Source: Commun Biol. 2023 Apr 24;6:449. doi: 10.1038/s42003-023-04839-6 (PMC10126163; doi:10.1038/s42003-023-04839-6)
Supplement: Supplementary file 2 — Reporting Summary [file 42003_2023_4839_MOESM2_ESM.pdf]

nature portfolio

Corresponding author(s): Kevin Tsia

Last updated by author(s): Apr 11, 2023

Reporting Summary

Nature Portfolio wishes to improve the reproducibility of the work that we publish. This form provides structure for consistency and transparency in reporting. For further information on Nature Portfolio policies, see our Editorial Policies and the Editorial Policy Checklist.

Statistics

For all statistical analyses, confirm that the following items are present in the figure legend, table legend, main text, or Methods section.

n/a	Confirmed	
☐	☒	The exact sample size (*n*) for each experimental group/condition, given as a discrete number and unit of measurement
☐	☒	A statement on whether measurements were taken from distinct samples or whether the same sample was measured repeatedly
☐	☒	The statistical test(s) used AND whether they are one- or two-sided<br>*Only common tests should be described solely by name; describe more complex techniques in the Methods section.*
☐	☒	A description of all covariates tested
☒	☐	A description of any assumptions or corrections, such as tests of normality and adjustment for multiple comparisons
☐	☒	A full description of the statistical parameters including central tendency (e.g. means) or other basic estimates (e.g. regression coefficient) AND variation (e.g. standard deviation) or associated estimates of uncertainty (e.g. confidence intervals)
☒	☐	For null hypothesis testing, the test statistic (e.g. *F*, *t*, *r*) with confidence intervals, effect sizes, degrees of freedom and *P* value noted<br>*Give P values as exact values whenever suitable.*
☒	☐	For Bayesian analysis, information on the choice of priors and Markov chain Monte Carlo settings
☐	☒	For hierarchical and complex designs, identification of the appropriate level for tests and full reporting of outcomes
☐	☒	Estimates of effect sizes (e.g. Cohen's *d*, Pearson's *r*), indicating how they were calculated

Our web collection on statistics for biologists contains articles on many of the points above.

Software and code

Policy information about availability of computer code

Data collection	Custom code and custom MATLAB code in supplementary software
Data analysis	Custom MATLAB code: https://doi.org/10.5281/zenodo.7787607

For manuscripts utilizing custom algorithms or software that are central to the research but not yet described in published literature, software must be made available to editors and reviewers. We strongly encourage code deposition in a community repository (e.g. GitHub). See the Nature Portfolio guidelines for submitting code & software for further information.

Data

Policy information about availability of data

All manuscripts must include a data availability statement. This statement should provide the following information, where applicable:

- Accession codes, unique identifiers, or web links for publicly available datasets
- A description of any restrictions on data availability
- For clinical datasets or third party data, please ensure that the statement adheres to our policy

A test dataset is uploaded in: https://doi.org/10.5281/zenodo.7787607
The complete data that support the findings of this study are available from the corresponding author upon reasonable request.

Field-specific reporting

Please select the one below that is the best fit for your research. If you are not sure, read the appropriate sections before making your selection.

☒ Life sciences ☐ Behavioural & social sciences ☐ Ecological, evolutionary & environmental sciences

For a reference copy of the document with all sections, see nature.com/documents/nr-reporting-summary-flat.pdf

Life sciences study design

All studies must disclose on these points even when the disclosure is negative.

Sample size	The sample sizes were determined by its total captured cell images and statistical analysis like effect size measures and correlation factor are calculated to ensure the sample size is sufficient.
Data exclusions	Images that are out-of-focus and blank with noise only are excluded.
Replication	Experiments for cell cycle analysis were repeated for three times, and the image data of lung cancer cell lines were obtained from multiple trails on different date.
Randomization	The cells were randomly extracted from the cell culture.
Blinding	For the lung cancer cell lines, the investigator was not blind to group allocation during the data acquisition and collection procedure because we need to provide the true label for single-cell analysis. For the cell cycle analysis, the investigator was blind during the data collection by high-speed cell flow, and the ground truth label was then determined from on-chip fluorescence detection.

Reporting for specific materials, systems and methods

We require information from authors about some types of materials, experimental systems and methods used in many studies. Here, indicate whether each material, system or method listed is relevant to your study. If you are not sure if a list item applies to your research, read the appropriate section before selecting a response.

Materials & experimental systems

n/a	Involved in the study	
☒	☐	Antibodies
☐	☒	Eukaryotic cell lines
☒	☐	Palaeontology and archaeology
☒	☐	Animals and other organisms
☒	☐	Human research participants
☒	☐	Clinical data
☒	☐	Dual use research of concern

Methods

n/a	Involved in the study	
☒	☐	ChIP-seq
☐	☒	Flow cytometry
☒	☐	MRI-based neuroimaging

Eukaryotic cell lines

Policy information about cell lines

Cell line source(s)	Breast cancer cell line: MDA-MB231; lung cancer cell lines: three adenocarcinoma cell lines (ADC, H358 (EGFR WT), HCC827 (EGFR exon 19 del) and H1975 (L858R and T790M)), two squamous cell carcinoma cell lines (SCC, H520 and H2170) and two small cell lung cancer cell lines (SCLC, H526 and H69) ; leukemia cell lines: THP-1 (TIB- 202TM) and Kasumi-1/ACC220 (CRL-2724TM).
Authentication	Cellular morphology are routinely checked during cell culture under light microscope prior to imaging experiments
Mycoplasma contamination	Preventing of mycoplasma contaminationwas done by adding antibiotic-antimycotic during cell culture
Commonly misidentified lines (See ICLAC register)	None were used.

March 2021

Flow Cytometry

Plots

Confirm that:

☒ The axis labels state the marker and fluorochrome used (e.g. CD4-FITC).

☒ The axis scales are clearly visible. Include numbers along axes only for bottom left plot of group (a 'group' is an analysis of identical markers).

☒ All plots are contour plots with outliers or pseudocolor plots.

☒ A numerical value for number of cells or percentage (with statistics) is provided.

Methodology

Sample preparation	MDA-MB231 cell line was purchased from American Type Culture Collection (ATCC) and cultured in100mm culture dish (Labserv), which were placed in a CO_2 incubator with 5% CO_2 under 37 °C. The full culture medium was ATCC modified RPMI-1640 (Gibco) supplemented with 10% fetal bovine serum (FBS) (Gibco) and 1% antibiotic–antimycotic (Gibco). Click-iT Plus EdU Flow Cytometry Assay Kits Alexa Fluor 488 and FxCycle PI/RNase Staining Solution were obtained from Invitrogen to define the ground-truth of the cell cycle stages (G1, S and G2 phases). The MDA-MB231 cell culture was firstly renewed for 8 mL medium mixed with 8 µL of 10 mM EdU staining solution. After 2-hour incubation, the cells were harvested by 0.25% Trypsin (Thermo Scientific) and washed by PBS with 1% BSA. The cells were then brought to protection from light for the following steps. The centrifuged cells were fixed by 100 µL Click-iT fixative (4% paraformaldehyde in PBS) and then permeabilized by the permeabilization and wash reagent (sodium azide) with 15-minute incubation. Next, 500 µL Click-iT Plus reaction cocktail was added into 100 µL cell suspension for 30 minutes under room temperature. After washing with 3 mL Click-iT permeabilization and wash reagent, the cell pellet was then mixed with 500 µL Click-iT permeabilization and wash reagent and 500 µL FxCycle PI/RNase staining solution for 30 minutes at room temperature. Lastly, after washing away the staining solution, PBS was added to make up a total volume of 1-3 mL cell suspension for the subsequent flow cytometry experiments.
Instrument	BD FACSAria™ III
Software	Collected by BD FACSDiva Software (Version 6.1.3), analysis by customized MATLAB
Cell population abundance	Over 50,000 cells were collected.
Gating strategy	The cells with a large (>2e5) or small (<0.5e5) PI intensity have been recognized to be cell aggregates and debris and gated out.

☒ Tick this box to confirm that a figure exemplifying the gating strategy is provided in the Supplementary Information.
